# PER2/P65-driven glycogen synthase 1 transcription in macrophages modulates gut inflammation and pathogenesis of rectal prolapse

**DOI:** 10.1016/j.jbc.2023.105219

**Published:** 2023-09-01

**Authors:** Zhao Ding, Wenhao Ge, Xiaodong Xu, Xi Xu, Shiming Wang, Jianfa Zhang

**Affiliations:** Center for Molecular Metabolism, Nanjing University of Science & Technology, Nanjing, China

**Keywords:** inflammatory, macrophage, *Per2*, rectal prolapse, inflammatory bowel disease

## Abstract

Rectal prolapse in serious inflammatory bowel disease is caused by abnormal reactions of the intestinal mucosal immune system. The circadian clock has been implicated in immune defense and inflammatory responses, but the mechanisms by which it regulates gut inflammation remain unclear. In this study, we investigate the role of the rhythmic gene *Period2* (*Per2*) in triggering inflammation in the rectum and its contribution to the pathogenesis of rectal prolapse. We report that *Per2* deficiency in mice increased susceptibility to intestinal inflammation and resulted in spontaneous rectal prolapse. We further demonstrated that PER2 was essential for the transcription of glycogen synthase 1 by interacting with the NF-κB p65. We show that the inhibition of *Per2* reduced the levels of glycogen synthase 1 and glycogen synthesis in macrophages, impairing the capacity of pathogen clearance and disrupting the composition of gut microbes. Taken together, our findings identify a novel role for *Per2* in regulating the capacity of pathogen clearance in macrophages and gut inflammation and suggest a potential animal model that more closely resembles human rectal prolapse.

Rectal prolapse is a condition where the rectum protrudes from the anus due to its displacement from the original position. While rectal prolapse can occur at any age, its incidence is increasing due to the aging of the population (1). The exact cause of rectal prolapse remains unclear, but it is commonly associated with chronic constipation and inflammatory bowel disease (2, 3). Severe intestinal inflammation is often observed in patients with rectal prolapse, and abnormal immune reactions of the intestinal mucosa play a crucial role in the pathogenesis of inflammatory bowel disease (4). Chronic inflammation can lead to tissue thickening, edema, and posterior tenesmus, which can provide a pathophysiological basis for the occurrence of rectal prolapse with inferior enteritis (5, 6).

Impaired mucosal immunity in the gut can lead to the development of intestinal inflammation, which is partly related to intestinal pathogens (7). The bacteria most commonly reported to be associated with rectal prolapse are enterohepatic *Helicobacter* species and *Citrobacter rodentium*, although theoretically any pathogen that causes colitis could predispose mice to rectal prolapse (8, 9). Rectal prolapse associated with microbial pathogens can occur in either immunocompetent or immunocompromised mice (10, 11). The body's first line of defense against pathogens is the innate immune system, of which a strong inflammatory response mediated by activation of the transcription factor NF-κB is an important component (12). In most cell types, NF-κB is represented primarily by the p65/p50 complex. Activation of NF-κB is triggered by a variety of microbial *via* pattern recognition receptors. The activated NF-κB complex enters the nucleus, binds to consistent sites of specific gene promoters, such as cytokines and various energy regulatory factors, and activates its expression (13). Gut macrophages play a vital role in defending against invading microbial pathogens by recognizing and phagocytizing them through the production of reactive oxygen species (ROS) (14). The glycogen of macrophages provides oxidation defense primarily through the production of NADPH (15). Lipopolysaccharide (LPS) treatment stimulates macrophages to synthesize glycogen, which then produces glucose-6-phosphate (G6P) through glycogen breakdown and further produces large amounts of NADPH *via* the pentose phosphate pathway. Macrophages need glycogen generation to store glycogen in their cells in order to effectively survive and kill bacteria when stimulated (16, 17). Blocking glycogen metabolism or inhibiting the NADPH oxidation process effectively inhibits inflammatory responses and promotes bacterial infection in a variety of mouse models (16, 18), and impaired macrophage function can cause intestinal microbial disorder and is closely linked to the occurrence of intestinal inflammation (19).

In mammals, the circadian clock has been implicated in immune defense and inflammatory responses (20). Mice treated with lethal doses of bacteria or bacterial products have shown significant circadian changes in survival rate (21). Some prospective studies have indicated that the clock genes are closely related to the function of immune cells. Disruptions in circadian rhythms can lead to increased gut inflammation (22). Understanding the relationship between gut inflammation and the circadian clock may have important implications for the prevention and treatment of various gastrointestinal disorders. More research is needed to fully elucidate the mechanisms through which the circadian clock regulates gut inflammation.

In this study, we investigated the potential role of circadian *Period2* (*Per2*) in rectal prolapse. PER2, a core clock protein localized in the nucleus, interacts with other clock proteins and transcription factors, forming regulatory complexes that control the expression of target genes involved in circadian rhythms and other cellular processes (23). Our findings revealed that mice deficient in Per2 spontaneously developed rectal prolapse, which was accompanied by inflammatory bowel disease and gut microbiota disorders. We demonstrated that PER2 interacts with p65 and affects the transcription of glycogen synthase 1 (*Gys1*). Loss of PER2 reduces the level of GYS1 in cultural macrophages, decreasing glycogen synthesis, and impairing the capacity of pathogen clearance. Our results suggest that PER2 is essential to the capacity of pathogen clearance in macrophages and that *Per2*-deficient mice represent a potential animal model of rectal prolapse.

## Results

### Spontaneous development of rectal prolapse in *Per2*-deficient mice

**Figure 1:**
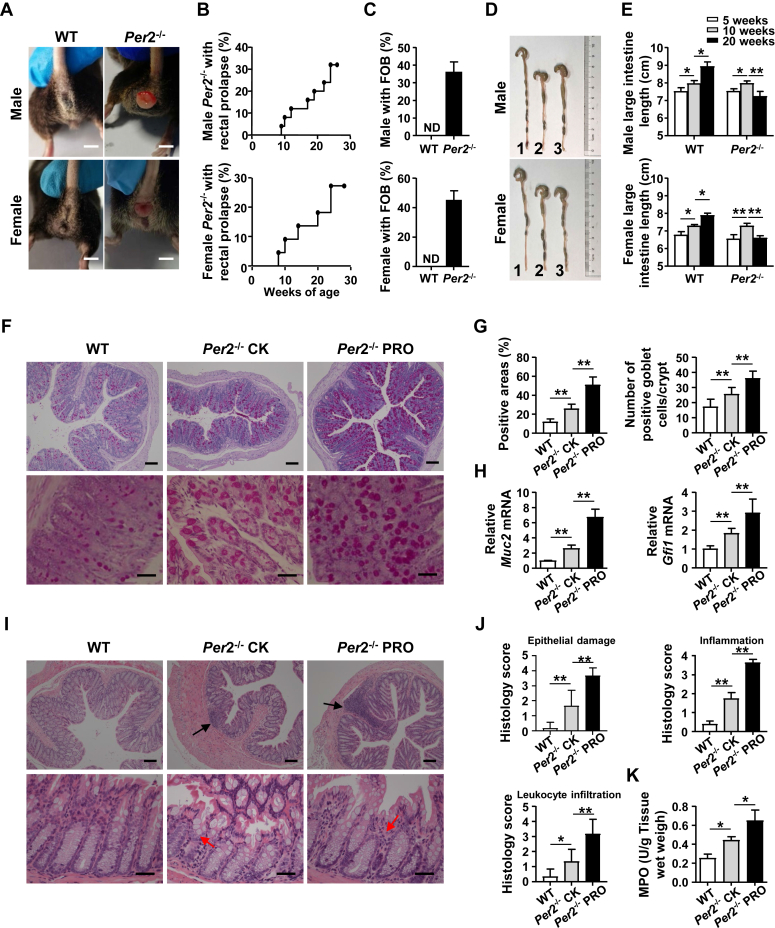
**Mice deficient in *Per2* spontaneously developed rectal prolapse.***A*, representative image of rectal prolapse in around 16-week-old male and female *Per2*^*−/−*^ mice. The scale bars represent 0.5 cm. *B*, frequency of male and female *Per2*^−/−^ mice with rectal prolapse. *C*, frequency of male and female WT and *Per2*^−/−^ mice with fecal occult blood (FOB). *D*, representative images of colon length in around 16-week-old male and female *Per2*^−/−^ mice (1: WT mice; 2: nonprolapsed *Per2*^−/−^ mice; 3: prolapsed *Per2*^−/−^ mice). *E*, colon length of male and female WT and *Per2*^−/−^ mice of different weeks of age. *F*, periodic acid–Schiff (PAS)-stained sections of rectums from 16-week-old WT mice, nonprolapsed and prolapsed *Per2*^−/−^ mice. The scale bars represent 100 μm (*top row*) or 10 μm (*bottom row*). *G*, assessment of positive areas and number of positive goblet cells/crypt of PAS-stained sections. *H*, the mRNA expression levels of *Muc2* and *Gfi1* in the rectums were measured by real-time PCR. *I*, H&E-stained sections of rectums from WT mice, nonprolapsed and prolapsed *Per2*^−/−^ mice. *Black arrow*, infiltration of immune cells; *red arrow*, aberrant crypt architecture. The scale bars represent 100 μm (*top row*) or 10 μm (*bottom row*). *J*, H&E-stained sections were scored for epithelial damage, inflammation, and leukocyte infiltration. Data are from one experiment representative of three independent experiments with similar results. *K*, rectums myeloperoxidase (MPO) activity of WT mice, nonprolapsed and prolapsed *Per2*^−/−^ mice. Data were shown as mean ± SD. Statistical analysis was performed by one-way ANOVA. Data are pooled from four independent experiments (*A*–*C*) or are from one experiment representative of three independent experiments with similar results (*D*–*K*; of n = 6–8 mice). ∗*p* < 0.05, ∗∗*p* < 0.01. BMDM, bone marrow–derived macrophage; MUC2, mucin 2; ND, not detected; Per2, Period2; *Per2*^−/−^ CK, nonprolapsed *Per2*^−/−^ mice; *Per2*^−/−^ PRO, prolapsed *Per2*^−/−^ mice; WT, WT mice.

We observed spontaneous rectal prolapse in both male and female *Per2*^−/−^ mice at approximately 2 to 8 months of age, with an incidence of 32% in males and 27% in females (Fig. 1, *A* and *B*), accompanied by fecal occult blood (Fig. 1*C*). *Per2*^−/−^ mice with rectal prolapse had reduced body weight but increased food and water intake, as well as a higher proportion of neutrophils in the blood (Fig. S1, *A*–*D*). At 16 weeks of age, *Per2*^−/−^ mice had a shorter length of large intestine than age- and sex-matched WT mice, regardless of prolapse (Fig. 1, *D* and *E*). Histological analysis showed that the rectum of *Per2*^−/−^ mice produced more mucin 2 (MUC2) protein, a major factor of mucosal immunity in the gut (Fig. 1*F*), but there was no reduction in goblet cell number (Fig. 1*G*). The expression of *Muc2* and goblet cell differentiation factor *Gfi1* in the rectum of *Per2*^−/−^ mice was also higher than that of WT mice (Fig. 1*H*), suggesting that the occurrence of rectal prolapse in *Per2*^−/−^ mice is not related to impairment of intestinal mucosal immunity. We observed more epithelial damage, inflammation, immune cell infiltration, and higher clinical scores in *Per2*^−/−^ mice than WT mice (Fig. 1, *I* and *J*). Increased myeloperoxidase activity also indicated increased granulocyte infiltration (Fig. 1*K*). MUC2 expression in the colon of *Per2*^−/−^ mice did not change compared to WT mice (Fig. S1, *E*–*G*), and colon inflammation only occurred when *Per2*^−/−^ mice developed rectal prolapse (Fig. S1, *H*–*K*). Collectively, these results suggested that susceptibility to rectal prolapse in *Per2*^−/−^ mice is closely associated with proctitis.

### Deficiency in *Per2* leads to inflammation preceding rectal prolapse

**Figure 2:**
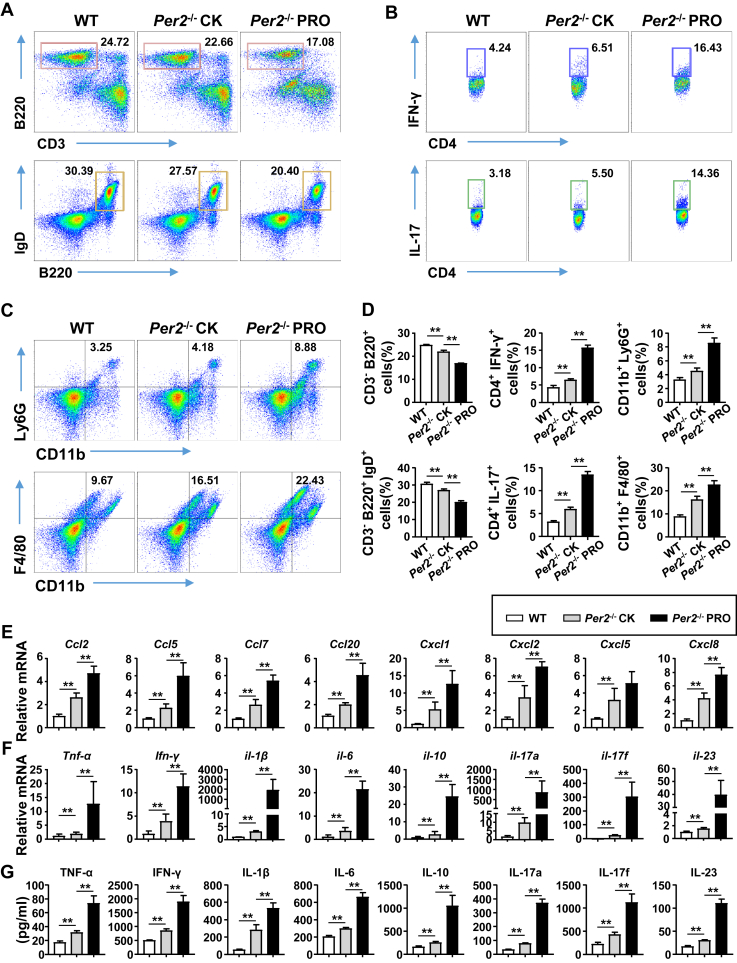
**Mice deficient in *Per2* developed inflammation in the rectum.***A*–*C*, flow cytometry of rLPLs isolated from 16-week-old WT mice, nonprolapsed and prolapsed *Per2*^−/−^ mice (n = 5–6). *A*, rectal LPLs stained with antibodies against CD3, B220, and IgD. Numbers adjacent to outlined areas indicate percent CD3^-^ B220^+^ cells (B lymphocyte cells) (*top row*) or CD3^-^ B220^+^ IgD^+^ cells (mature IgD^+^ B cells) (*bottom row*). *B*, rectal LPLs stained with antibodies against CD3, CD4, IFN-γ, and IL-17. Numbers adjacent to outlined areas indicate percent CD4^+^ IFN-γ^+^ cells (Th1 cells) (*top row*) or CD4^+^ IL-17^+^ cells (Th17 cells) (*bottom row*). *C*, rectal LPLs stained with antibodies against CD11b, Ly6G, and F4/80. Numbers indicate percent CD11b^+^ Ly6G^+^ cells (neutrophils) (*top row*) or CD11b^+^ F4/80^+^ cells (macrophages) (*bottom row*). *D*, frequency of cells as in *A*–*C* in WT mice, nonprolapsed and prolapsed *Per2*^−/−^ mice. *E*, the mRNA expression levels of chemokines (*Ccl2*, *Ccl5*, *Ccl7*, *Ccl20*, *Cxcl1*, *Cxcl2*, *Cxcl5*, and *Cxcl8*) in the rectums were measured by real-time PCR. *F*, the mRNA expression levels of cytokines (*Tnf-α*, *Ifn-γ*, *il-1β*, *il-6*, *il-10*, *il-17a*, *il-17f*, and *il-23*) in the rectums were measured by real-time PCR. *G*, ELISA of cytokines as in *F* in explant cultures of rectums from WT mice, nonprolapsed and prolapsed *Per2*^−/−^ mice (n = 5–8). Data were shown as mean ± SD. Statistical analysis was performed by one-way ANOVA. ∗*p* < 0.05, ∗∗*p* < 0.01. IgD, immunoglobulin D; Per2, Period2; rLPL, rectal lamina propria lymphocyte.

In order to determine the major factor responsible for inducing rectal prolapse in *Per2*^−/−^ mice, we enriched rectal lamina propria (LP) lymphocytes from the rectal mucosa of 16-week-old WT mice, nonprolapsed and prolapsed *Per2*^−/−^ mice, and analyzed the types of infiltrating cells. We observed that the proportion of B lymphocytes and mature immunoglobulin D+ B cells were lower in normal *Per2*^−/−^ mice than in WT mice (Fig. 2, *A* and *D*). The increased proportion of Th1 and Th17 cells in CD4+ T cells suggested intense inflammation occurred in the rectum of *Per2*^−/−^ mice (Fig. 2, *B* and *D*). The number of neutrophils and macrophages was increased, indicating severe proctitis in *Per2*^−/−^ mice (Fig. 2, *C* and *D*), and the results of immunohistochemistry were consistent with flow cytometry (Fig. S2). These changes in immune cells were more pronounced in prolapsed *Per2*^−/−^ mice. Similar changes in immune cells in the spleen and mesenteric lymph nodes (MLNs) were observed in nonprolapsed and prolapsed *Per2*^−/−^ mice (Fig. S3, *A*–*C* and *G* and *H*). Natural killer cells and CD8+ T cells were increased in the spleen and MLN, and blood neutrophils and macrophages were also increased (Fig. S3*D*–*F*). Real-time PCR analysis showed that genes encoding chemokines (*Ccl2*, *Ccl5*, *Ccl7*, *Ccl20*, *Cxcl1*, *Cxcl2*, *Cxcl5*, and *Cxcl8*) and cytokines (*Tnf-α*, *Ifn-γ*, *il-1β*, *il-6*, *il-10*, *il-17a*, *il-17f*, and *il-23*) were much higher in the rectum of *Per2*^−/−^ mice (Fig. 2, *E* and *F*). ELISA results of cytokines were consistent with the mRNA data (Fig. 2*G*). Together, these results reveal that a deficiency in *Per2* leads to intense inflammation in the rectum prior to rectal prolapse.

### Alterations in gut microbiota and susceptibility to infection in *Per2*^−/−^ mice

**Figure 3:**
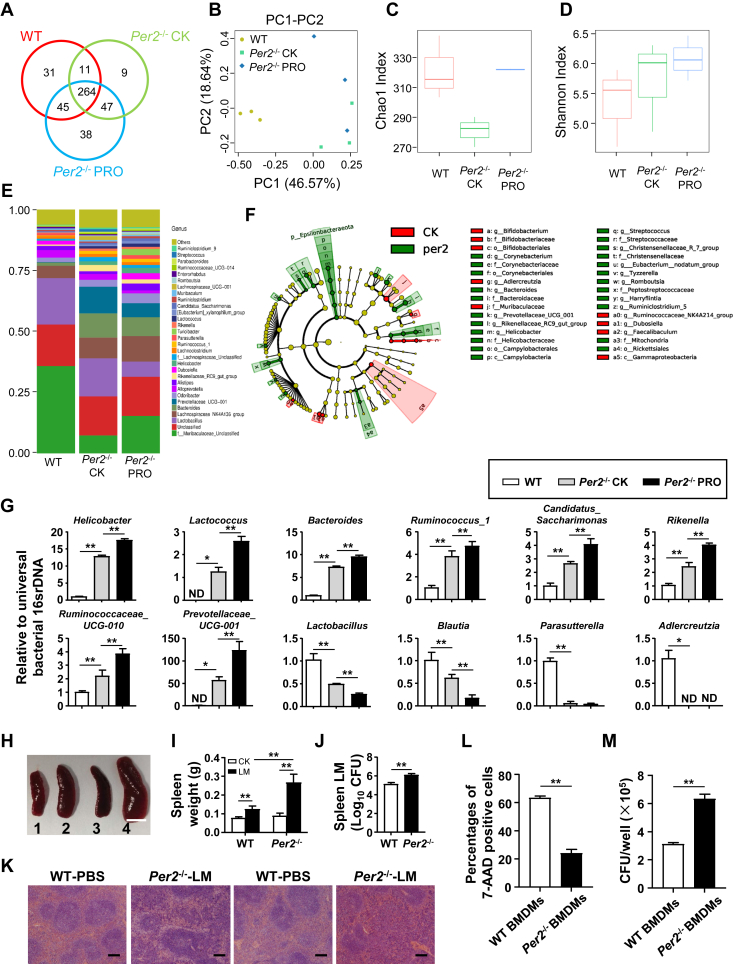
**Mice deficient in *Per2* showed decreased ability to clear pathogens and altered gut microbiota composition.***A*–*F*, results from 16S ribosomal DNA sequencing of fecal pellets from 16-week-old WT mice, nonprolapsed and prolapsed *Per2*^−/−^ mice (n = 3). *A*, venn diagram based on operational taxonomic units (OTU). *B*, Chao 1 index. *C*, Shannon index. *D*, principal co-ordinate analysis (PCoA) of the fecal microbiota. *E*, relative abundance of genus-classified gut microbiota. *F*, linear discriminant analysis (LDA) effect size analysis (LEfSe) was calculated between WT mice and nonprolapsed *Per2*^−/−^ mice. *G*, the relative abundance of several major microflora in the gut microflora were measured by real-time PCR (n = 3). *H*–*K*, 6-week-old WT and *Per2*^−/−^ mice were intravenously infected with *Listeria monocytogenes* (LM), and the results were analyzed 3 days later (n = 6–8). *H*, representative images of spleens in WT and *Per2*^−/−^ mice infected without or with LM (1: WT mice; 2: nonprolapsed *Per2*^−/−^ mice; 3: prolapsed *Per2*^−/−^ mice). Data are representative of three independent experiments with similar results. The scale bars represent 0.5 cm. *I*, spleen weight. *J*, colony-forming units (CFUs) were assayed in spleens. *K*, H&E-stained sections of spleens. The scale bars represent 100 μm. *L*, the percentages of 7-AAD–positive cells in WT BMDMs and *Per2*^−/−^ BMDMs was counted by flow cytometry. *M*, CFUs were assayed in BMDMs. Data are pooled from three independent experiments. Data were shown as mean ± SD. Statistical analysis was performed by Student’s *t* test, one-way ANOVA and two-way ANOVA. ∗*p* < 0.05, ∗∗*p* < 0.01. 7-AAD, 7-aminoactinomycin D; BMDM, bone marrow–derived macrophage; Per2, Period2.

To examine the composition of gut microbiota in 16-week-old WT mice, nonprolapsed and prolapsed *Per2*^−/−^ mice, we conducted 16S ribosomal DNA sequencing of the V3-V4 region of fecal pellets. Cluster analysis of operational taxonomic units (OTUs) and principal co-ordinate analysis based on UniFrac distance revealed a significant difference in the microbial community structure between *Per2*^−/−^ and WT mice (Fig. 3, *A* and *B*). While all groups of mice had a similar number of gut microbial species, the microbial diversity varied (Fig. 3, *C* and *D*). Specifically, several species from the *Helicobacter*, *Lactococcus*, *Bacteroides*, and *Ruminococcus* families were increased in *Per2*^−/−^ mice, whereas the percentage of *Lactobacillus* family members was reduced (Fig. 3*E*). Linear discriminant analysis effect size analysis demonstrated significant effects on species classification in WT and *Per2*^−/−^ mice (Fig. 3*F*), which was confirmed by real-time PCR analysis (Fig. 3*G*). Notably, the gut microbiota of *Per2*^−/−^ mice was altered even without prolapse, indicating that the alteration of gut microbiota occurred prior to prolapse in *Per2*^−/−^ mice. Changes in gut microbiota are associated with dysfunction of innate immunity, which in turn affects the early clearance of bacterial infections (24). To investigate the cause of the gut microbiota changes in *Per2*^−/−^ mice, we evaluated the susceptibility of 6-week-old WT and *Per2*^−/−^ mice to *Listeria monocytogenes* (LMs) infection *in vivo*. Our results showed that *Per2*^−/−^ mice had increased volume and weight gain of the spleen and higher abundance of LM in the spleen than WT mice (Fig. 3, *H*–*J*). Moreover, H&E staining revealed more severe immune response and damage in the spleen of *Per2*^−/−^ mice, confirming their increased susceptibility to LM infection in the absence of *Per2* (Fig. 3*K*). We then evaluated the phagocytosis of *Per2*^−/−^ bone marrow–derived macrophages (BMDMs), considering that macrophage clearance plays an important role in the early stages of LM infection and that BMDMs is accepted as a substitute for tissue-resident macrophages in the assessment of phagocytosis activity (25, 26). Flow cytometry analysis and killing activity assay of macrophages demonstrated a reduced ability of *Per2*^−/−^ BMDMs to phagocytize and kill *Escherichia coli* (Fig. 3, *L* and *M*). Our findings indicate that *Per2* deficiency not only impairs pathogen clearance in macrophages but also alters gut microbiota, providing insights into the mechanisms underlying the pathogenesis of prolapse.

### *Per2* deficiency leads to reduced glycogenesis in macrophages

**Figure 4:**
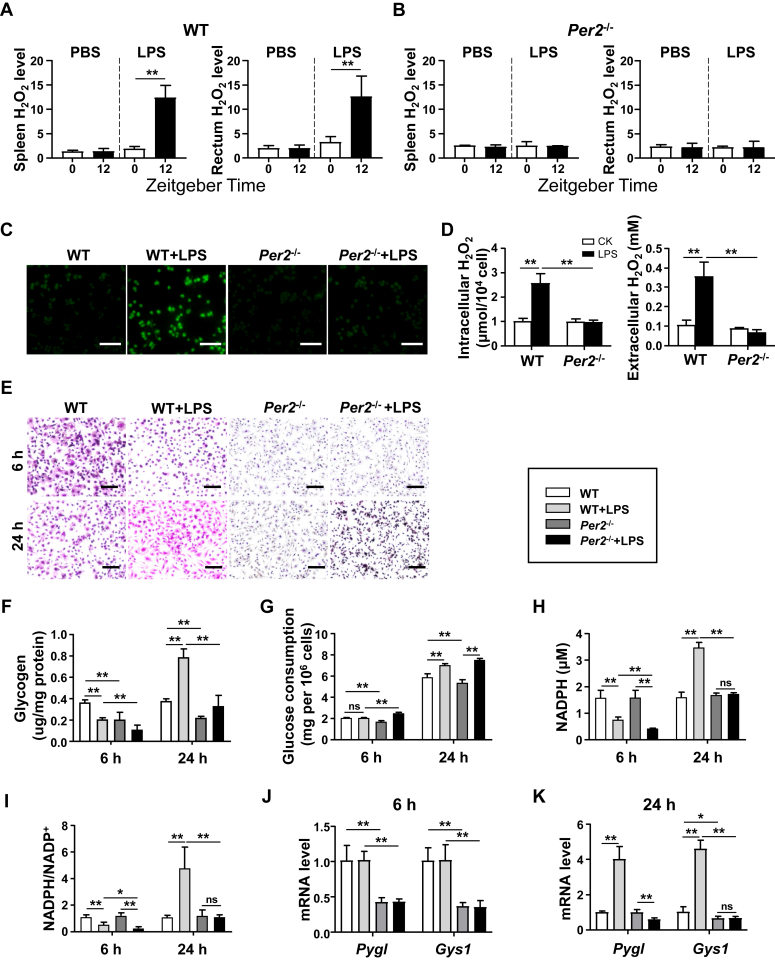
**Mice deficient in *Per2* showed impaired glycogenesis under LPS stimulation.***A* and *B*, 6-week-old WT and *Per2*^−/−^ mice were challenged intraperitoneally with 5 mg LPS/kg in ZT0 and ZT12 (n = 6–8). Spleen and rectum were separated for hydrogen peroxide (H_2_O_2_) determination 6 h later. *A*, H_2_O_2_ levels in the spleen (*left*) and rectum (*right*) of WT mice. *B*, H_2_O_2_ levels in the spleen (*left*) and rectum (*right*) of *Per2*^−/−^ mice. *C* and *D*, the H_2_O_2_ levels of BMDMs from WT and *Per2*^−/−^ mice were detected after LPS (100 ng/ml) stimulation for 24 h. *C*, representative fluorescence image of intracellular H_2_O_2_ labeled by H2DCF-DA (*green*) in BMDMs. The scale bars represent 100 μm. *D*, BMDMs intracellular (*left*) and extracellular (*right*) H_2_O_2_ concentrations. *E*–*K*, intracellular glycogen levels in BMDMs were observed and detected by PAS staining (The scale bar represents 100 μm) (*E*) and colorimetric assay (*F*) after LPS stimulation for 6 and 24 h. *G*, BMDMs were stimulated with LPS for 6 and 24 h and analyzed for glucose consumption. *H* and *I*, intracellular NADPH concentration (*H*) and NADPH/NADP^+^ ratio (*I*) of BMDMs stimulated with LPS for 6 and 24 h were analyzed. *J* and *K*, relative mRNA expression of *Pygl* and *Gys1* of BMDMs stimulated with LPS for 6 (*J*) and 24 h (*K*) were determined by real-time PCR. Data are pooled from three independent experiments. Data were shown as mean ± SD. Statistical analysis was performed by two-way ANOVA. ∗*p* < 0.05, ∗∗*p* < 0.01. BMDM, bone marrow–derived macrophage; *Gys1*, glycogen synthase 1; LPS, lipopolysaccharide; PAS, periodic acid–Schiff; Per2, Period2.

In order to identify the effect of *Per2* on the pathogen clearance ability of macrophages, we developed a model of LPS-infected mice. LPS stimulation resulted in significant production of hydrogen peroxide (H_2_O_2_) in the spleen and rectum of WT mice (Fig. 4*A*), while such production was not observed in *Per2*^−/−^ mice (Fig. 4*B*). H_2_O_2_ plays a crucial role in macrophage-mediated defense against bacterial infections and can kill phagocytosed pathogens directly or indirectly. We also found that in LM infection models, spleen and peritoneal macrophages of *Per2*^−/−^ mice produced lower levels of H_2_O_2_ than WT mice (Fig. S4, *A* and *B*). In isolated BMDMs from WT and *Per2*^−/−^ mice, *Per2*^−/−^ BMDMs produced less H_2_O_2_ compared to WT BMDMs after LPS stimulation (Fig. 4, *C* and *D*). It is well known that H_2_O_2_ produced by macrophages activated by LPS is derived from the oxidation of NADPH, which is produced by the pentose phosphate pathway during glycogenolysis (16). We then discovered that the amount of glycogen stored in *Per2*^−/−^ BMDMs was lower than that in WT BMDMS at rest (Fig. 4*E*). After 6 h of LPS stimulation, the glycogen content of BMDMs decreased in both groups. However, 24 h after LPS stimulation, we observed a significant increase in glycogen in WT BMDMs, but not in *Per2*^−/−^ BMDMs (Fig. 4*F*). After LPS stimulation, *Per2*^−/−^ BMDMs consumed more glucose than WT BMDMs, but at rest *Per2*^−/−^ BMDMs consumed less glucose, which is consistent with the low glycogen storage in *Per2*^−/−^ BMDMs (Fig. 4*G*). Moreover, lower levels of NADPH and NADPH/NADP^+^ ratios were observed in *Per2*^−/−^ BMDMs than WT BMDMs at both 6 and 24 h after LPS stimulation (Fig. 4, *H* and *I*). We subsequently examined the key genes involved in glycogenesis in macrophages. *Per2*^−/−^ BMDMs showed reduced expression of *Pygl* and *Gys1* compared with WT BMDMs, both at 6 h or 24 h after LPS stimulation (Fig. 4, *J* and *K*). At 24 h after LPS challenge, *Per2*^−/−^ BMDMs had lower levels of nitric oxide and mitochondrial ROS than WT BMDMs (Fig. S5). Consistently, *Per2*^−/−^ mice showed lower glycogen content in liver and muscle than that of WT mice, as well as lower expression of glycogen synthase (Fig. S4, *C*–*E*). The expression of *Gys1* in spleen, rectum, and peritoneal macrophages of *Per2*^−/−^ mice was also lower than that of WT mice after LPS stimulation (Fig. S4, *F*–*H*). Taken together, these results suggest that *Per2* deficiency leads to decreased expression of *Gys1* and reduced glycogenesis.

### PER2 physically binds to p65

**Figure 5:**
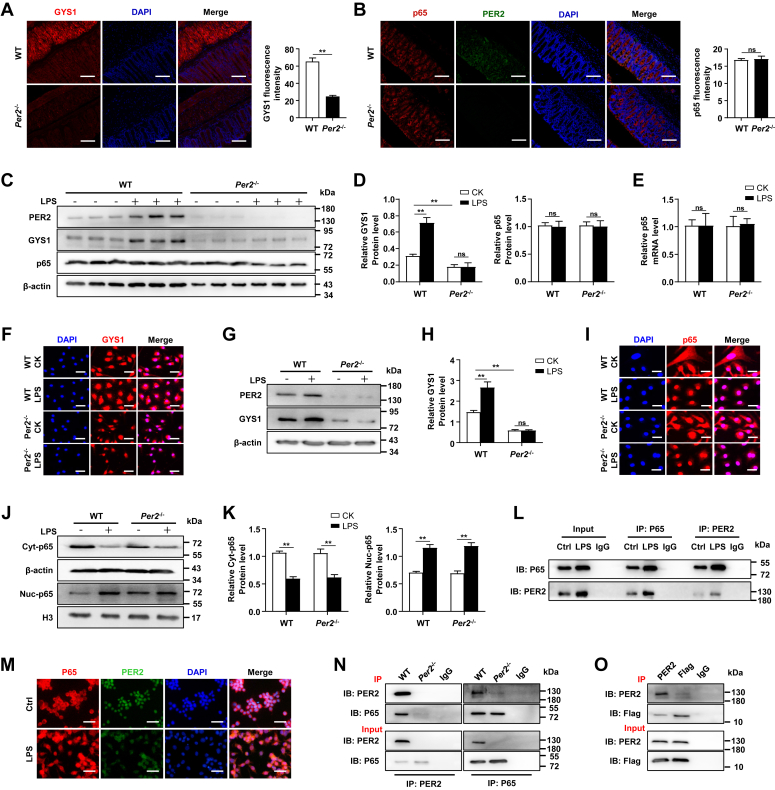
**PER2 affects the expression of GYS1 by binding to p65.***A* and *B*, representative images with immunostaining and statistical analysis of the fluorescence intensities of GYS1 (*A*), p65, PER2 (*B*), and DAPI on rectum sections of around 16-week-old WT and nonprolapsed *Per2*^−/−^ mice. The scale bar represents 20 μm. *C*–*E*, 6-week-old WT and *Per2*^−/−^ mice were challenged intraperitoneally with 5 mg LPS/kg. *C*, PER2, GYS1, and p65 expression in rectum was determined by Western blot. *D*, fluorescence intensity of GYS1 (*left*) and p65 (*right*) were analyzed. *E*, relative mRNA expression of p65 was analyzed by real-time PCR. *F*–*K*, BMDMs from WT and *Per2*^−/−^ mice were stimulated with LPS for 24 h. GYS1 location was analyzed by confocal microscope (The scale bar represents 20 μm) (*F*), PER2 and GYS1 expression were determined by Western blot (*G*), fluorescence intensity of GYS1 was analyzed (*H*). P65 location was analyzed by confocal microscope (The scale bar represents 10 μm) (*I*), nuclear-p65 and cytoplasm-p65 expression were determined by Western blot (*J*), fluorescence intensity of nuclear-p65 and cytoplasm-p65 were analyzed (*K*). *L*, representative coimmunoprecipitation results of PER2 and p65 interaction from nuclear extracts of BMDMs from WT mice. *M*, representative immunofluorescence images of p65 (CY3-labeled) and PER2 (Alexa Fluor 488–labeled) in BMDMs from WT mice. The scale bar represents 50 μm. *N*, immunoassay of lysates of WT and *Per2*^−/−^ rectal LPLs subjected to immunoprecipitation (IP) with anti-PER2 or anti-p65 and immunoblot analysis (IB) with anti-PER2 or anti-p65. *O*, immunoassay of lysates of NIH3T3 cells transfected with plasmids encoding Flag-p65, followed by IP with anti-PER2, anti-Flag or the control antibody immunoglobulin G (IgG) and IB with anti-PER2 or anti-Flag; input (*below*), immunoblot analysis of the samples above without immunoprecipitation. Data are pooled from three independent experiments. Data were shown as mean ± SD. Statistical analysis was performed by Student’s *t* test and two-way ANOVA. ∗*p* < 0.05, ∗∗*p* < 0.01. BMDM, bone marrow–derived macrophage; DAPI, 4',6-diamidino-2-phenylindole; *Gys1*, glycogen synthase 1; LPS, lipopolysaccharide; Per2, Period2.

Previous studies have demonstrated that p65 can bind to the promoter of *Gys1* to regulate its expression (27). To gain insight into the correlation between *Per2* and *Gys1* expression, we investigated the level of p65, a key transcription factor of *Gys1*, in rectal tissue of WT and *Per2*^−/−^ mice. Immunofluorescence analysis using anti-GYS1, anti-P65, and anti-PER2 antibodies revealed higher levels of GYS1 in the rectum of WT mice than in *Per2*^−/−^ mice (Fig. 5*A*), while p65 levels did not differ (Fig. 5*B*). Furthermore, GYS1 expression was elevated in the rectum of WT mice after LPS treatment, while no increase was observed in *Per2*^−/−^ mice (Fig. 5*C*). There was no difference in the level of p65 protein between the two groups (Fig. 5, *C* and *D*), and the p65 gene expression also remained consistent at the protein level (Fig. 5*E*). *In vitro*, the absence of *Per2* downregulated GYS1 expression in LPS-treated BMDMs (Fig. 5*F*). The observation of cellular immunofluorescence analysis was consistent with results from the Western blot assay, which showed an increased GYS1 protein level in WT BMDM after LPS stimulation, but no change in *Per2*^−/−^ BMDMs (Fig. 5, *G* and *H*). Similarly, the entry of p65 into the nucleus after LPS treatment was observed by cellular immunofluorescence, and the absence of *Per2* did not affect this process (Fig. 5*I*). Western blot assay showed that the cytoplasmic and nuclear protein levels of p65 in the two groups of BMDMs were consistent after LPS treatment (Fig. 5, *J* and *K*). Co-immunoprecipitation (co-IP) of PER2 and p65 in nucleoprotein extracts from WT BMDMs showed that PER2 directly bound to p65 in the nucleus, and the binding efficiency was more obvious after LPS stimulation (Fig. 5*L*). Immunofluorescence images of p65 and PER2 further confirmed the above binding (Fig. 5*M*). To determine whether this interaction occurs endogenously in the rectal mucosa, we performed co-IP experiments with rectal LP lymphocytes from WT and *Per2*^−/−^ mice. We found that PER2 coimmunoprecipitates with p65 and vice versa (Fig. 5*N*). We then evaluated this interaction by transient transfection of Flag-tagged p65 (Flag-p65) into NIH3T3 cells. Immunoprecipitation with mutual antibodies confirmed protein–protein interactions between PER2 and p65 (Fig. 5*O*). All these results indicate that PER2 and p65 physically interacted.

### LPS stimulation enhances the formation of the PER2–P65 complex on the *Gys1* promoter

**Figure 6:**
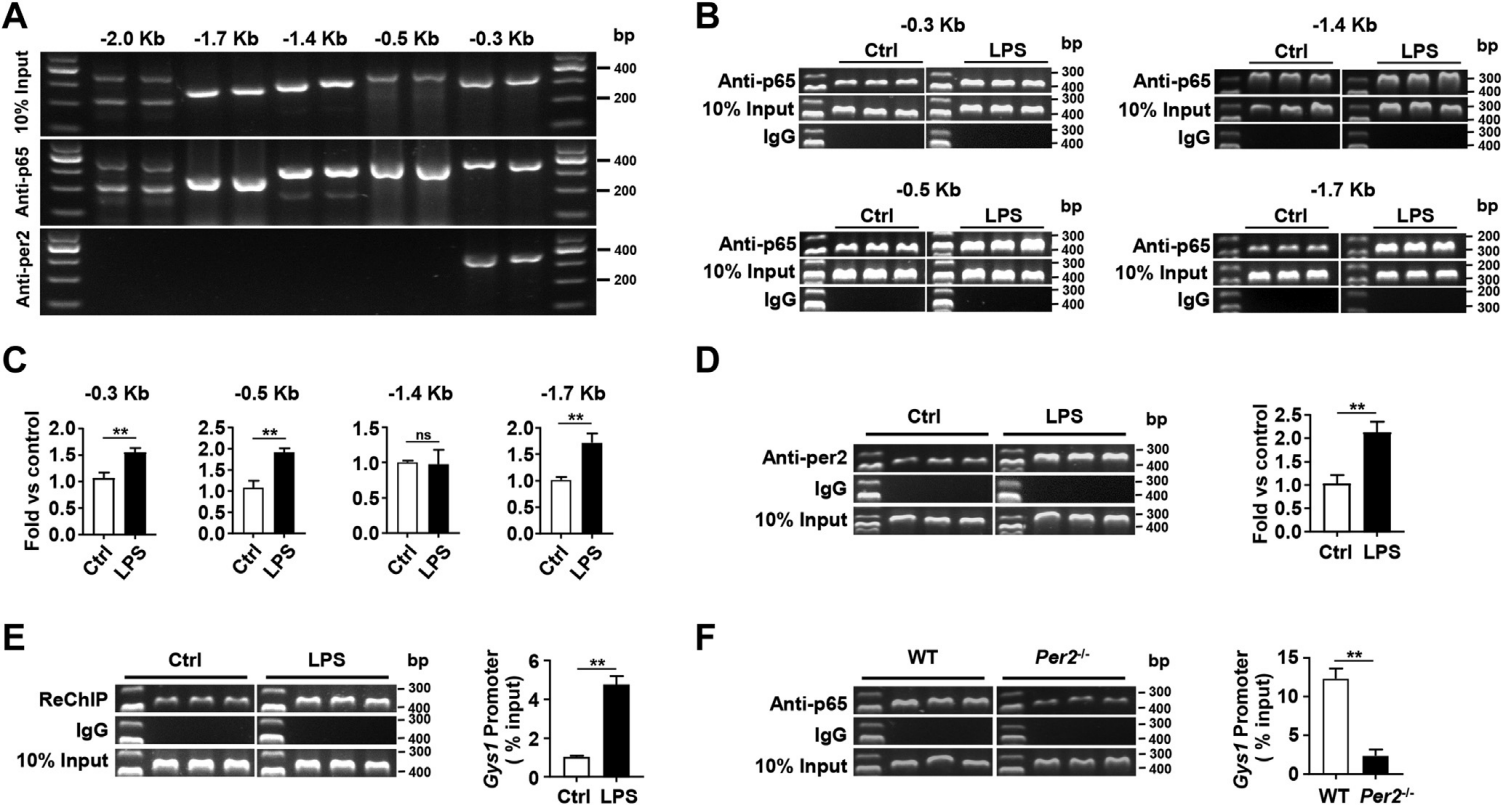
**The binding of PER2 and p65 is enhanced on the *Gys1* promoter after LPS stimulation.***A*–*E*, BMDMs from WT mice were stimulated with or without LPS for 24 h. P65 and PER2 enrichment around the promoter of *Gys1* were analyzed by ChIP-PCR. *A*, ChIP results of p65 and PER2 binding in different regions of the *Gys1* promoter without LPS stimulation. *B*, ChIP results of p65 binding in the −0.3 Kb, −0.5 Kb, −1.4 Kb, and −1.7 Kb region of the *Gys1* promoter with or without LPS stimulation. *C*, ChIP-qPCR were used to *Gys1* quantitative detection. *D*, ChIP results of PER2 binding in the −0.3 Kb region of the *Gys1* promoter with or without LPS stimulation (*left*). ChIP-qPCR were used to *Gys1* quantitative detection (*right*). *E*, ChIP-reChIP results of p65 and PER2 binding in *Gys1* promoter (*left*). ChIP-qPCR were used to *Gys1* quantitative detection and total genomic DNA was used as input (*right*). *F*, ChIP results of p65 binding in *Gys1* promoter after LPS stimulation of BMDMs from WT and *Per2*^−/−^ mice (*left*). ChIP-qPCR were used to *Gys1* quantitative detection (*right*). Data are pooled from three independent experiments. Data were shown as mean ± SD. Statistical analysis was performed by Student’s *t* test. ∗*p* < 0.05, ∗∗*p* < 0.01. BMDM, bone marrow–derived macrophage; ChIP, chromatin immunoprecipitation; *Gys1*, glycogen synthase 1; LPS, lipopolysaccharide; Per2, Period2; qPCR, quantitative PCR.

Bioinformatic analysis using the UCSC Genome Browser and JASPAR identified multiple consistent p65 binding elements on the *Gys1* promoter, and we designed five primer pairs for the *Gys1* promoter. Chromatin immunoprecipitation (ChIP)-PCR assays showed that p65 directly bound to multiple regions of the *Gys1* promoter in BMDMs, while PER2 was linked to the −0.3 Kb region of the *Gys1* promoter *via* p65 (Fig. 6*A*). Additionally, the binding efficiency of different regions of the *Gys1* promoter and p65 was inconsistent after LPS stimulation (Fig. 6, *B* and *C*). We then focused on the binding of p65 and PER2 to the −0.3 Kb region of the *Gys1* promoter. ChIP-qPCR results showed that the binding efficiency of PER2 to the *Gys1* promoter was enhanced after LPS stimulation (Fig. 6*D*). The ChIP-reChIP assay further demonstrated that p65 and PER2 formed a complex and then bound to the Gys1 promoter, and LPS stimulation enhanced this effect (Fig. 6*E*). ChIP experiments in LPS-stimulated WT and *Per2*^−/−^ BMDMs indicated that *Per2* deficiency weakened p65 and *Gys1* promoter binding (Fig. 6*F*), and it also led to a decrease of p65 binding to *tnf-α*, *il-1β*, and *il-6* promoters (Fig. S6). These findings suggest that *Per2* deficiency decreases *Gys1* transcription by weakening the binding of p65 to the *Gys1* promoter.

### Inhibition of *Per2* reduced glycogenesis in cultured macrophages

**Figure 7:**
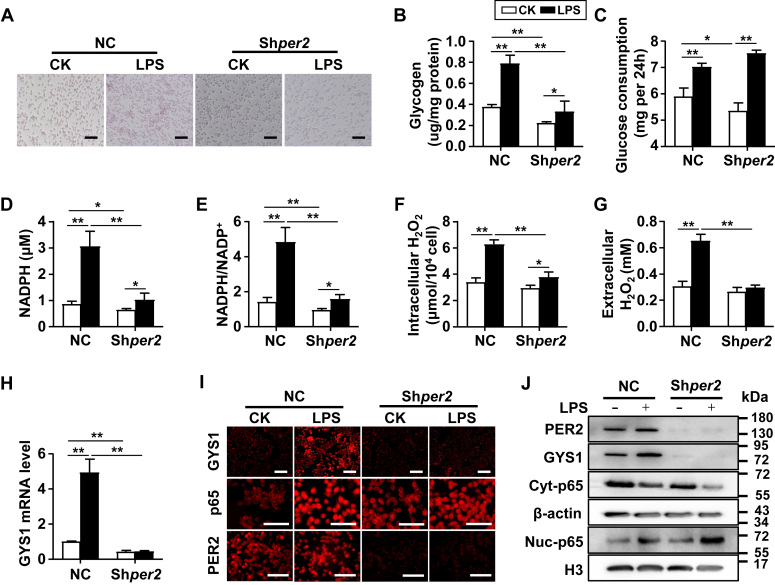
***Per2* knockdown attenuates *Gys1* expression in Raw264.7.***A*–*J*, Raw264.7 cells were infected with negative control (NC) shRNA lentiviruses or *Per2* shRNA (Sh*Per2*) lentiviruses then stimulated with LPS for 24 h. Intracellular glycogen levels in Raw264.7 cells were observed and detected by PAS staining (The scale bar represents 100 μm) (*A*) and colorimetric assay (*B*). Glucose consumption (*C*), intracellular NADPH concentration (*D*), NADPH/NADP^+^ ratio (*E*), intracellular (*F*), and extracellular (*G*) H_2_O_2_ concentrations of Raw264.7 cells were analyzed. *H*, relative mRNA expression of *Gys1* were determined by real-time PCR. *I*, representative immunofluorescence image of GYS1, p65, and PER2 in Raw264.7 cells. The scale bar represents 100 μm. *J*, PER2, GYS1, nuclear-p65, and cytoplasm-p65 expression were determined by Western blot. Data are pooled from three independent experiments. Data were shown as mean ± SD. Statistical analysis was performed by two-way ANOVA. ∗*p* < 0.05, ∗∗*p* < 0.01. *Gys1*, glycogen synthase 1; H_2_O_2_, hydrogen peroxide; LPS, lipopolysaccharide; PAS, periodic acid–Schiff; Per2, Period2.

We then investigated whether downregulation of *Per2* expression in macrophages inhibited GYS1 expression and reduced glycogen synthesis using *Per2*-specific shRNA lentiviruses in Raw264.7 cells. The transfection efficiency of lentivirus infection was confirmed by green fluorescence (Fig. S7*A*). The expression level of *Per2* mRNA was significantly decreased in Sh*per2*-treated cells (Fig. S7*B*). Methyl thiazolyl tetrazolium assay showed that the loss of *Per2* did not affect the proliferation of macrophages, either in resting state or after LPS stimulation (Fig. S7*C*). periodic acid–Schiff staining showed that sh*per2* treatment reduced glycogen storage in Raw264.7 cells and decreased glycogen production after LPS stimulation (Fig. 7, *A* and *B*). Furthermore, *Per2* knockdown decreased NADPH production and NADPH/NADP^+^ ratio after LPS stimulation, which led to a decrease in intracellular and extracellular H_2_O_2_ production (Fig. 7, *C*–*G*). *Per2* knockdown also reduced the levels of mitochondrial ROS and pyruvate, as well as the expression of inflammatory factors (Fig. S7, *D*–*F*). The *Per2*-specific shRNA reversed the trend of increased *Gys1* expression induced by LPS stimulation in macrophages (Fig. 7*H*). Cellular immunofluorescence analysis demonstrated that sh*per2* treatment reduced the expression of PER2 and GYS1, but did not affect p65 (Fig. 7*I*). Western blotting of cell lysates using anti-PER2, anti-GYS1, and anti-p65 confirmed that the expression of PER2 and GYS1 was reduced, and the entry of p65 into the nucleus was not affected (Fig. 7*J*). Taken together, these results indicate that interfering with *Per2* decreased the expression of GYS1, and consequently, reduced glycogen synthesis in macrophages.

## Discussion

Rectal prolapse is a serious problem in the aging population, and its prevalence has increased in recent years (28). For a long time, rectal prolapse has been recognized as a symptom that needs to be focused on discovering its underlying pathology or disease (29). However, there is no suitable mouse model of spontaneous rectal prolapse matching human rectal prolapse. In this study, we found that *Per2*-null mice had a decreased capacity for pathogen clearance, resulting in a progressive disturbance of the gut microbiome that eventually led to the natural occurrence of rectal prolapse. Our results provide a previously unknown PER2-p65–GYS1 pathway that regulates GYS1 expression and GYS1-mediated macrophage immunity and suggest an animal model that matches human rectal prolapse disease.

Our previous studies have found that aging leads to a decrease in the expression of the rhythmic gene *Per2* in the liver of mice (30). Aging also led to an impairment of *Per2* expression in the rectal tissues of mice (Fig. S8). Aging is a complex biological process that affects the digestive system, increasing the risk of gastrointestinal disorders, including rectal prolapse (31). Gut inflammation plays a crucial role in the development of rectal prolapse. Chronic inflammation can cause the weakening of the anal muscles and connective tissues, which can lead to anal prolapse. Inflammatory bowel disease, including Crohn's disease and ulcerative colitis, is associated with a higher risk of rectal prolapse. These conditions cause chronic inflammation in the digestive tract, which can lead to anal prolapse (3, 11, 32). Several studies have shown that the circadian clock plays a crucial role in regulating gut inflammation (33, 34). In particular, the circadian protein CLOCK can upregulate NF-κB–mediated transcription, and its overexpression correlates with an increase in specific phosphorylated and acetylated transcriptionally active forms of p65 (35). It is known that increased expression of NF-κB p65 in the liver and colon in *Per2*-deficient mice (36). In this study, we observed that PER2 and p65 form complexes, but the absence of PER2 does not affect the protein level and the phosphorylation of p65 (Fig. S9). In prolapsed *Per2*^−/−^ mice, the impaired capacity for pathogen clearance in macrophages causes an increase in T cells, leading to the production of pro-inflammatory cytokines and the development of inflammation.

Although rectal prolapse is a common occurrence in immunosuppressed mice, we have identified rectal prolapse lesions in *Per2*^−/−^ mice that are associated with chronic inflammation and gut microbial disturbance. The rectal prolapse observed in *Per2*^−/−^ mice differs from that seen in other mice that develop rectal prolapse, as the inflammatory lesions are limited to rectal tissue, with little or no inflammation in the cecum or colon tissue. Conversely, immunocompromised mice that develop rectal prolapse typically exhibit severe colitis (37). Given the limitations of the lesions, we believe that the initial injury originates from the distal rectal epithelial tissue. In comparison to the proximal colon, the distal rectum is more susceptible to pathogen exposure, indicating that PER2 plays a unique role in defending rectal tissue against pathogen invasion, and the lack of *Per2* renders the distal rectal tissue susceptible to pathogen infection.

In *Per2*^−/−^ mice with rectal prolapse, there is a marked proinflammatory response in the rectal tissue. The number of goblet cells did not decrease and more mucous proteins were expressed in the rectal prolapse of *Per2*^−/−^ mice, suggesting that the rectal prolapse in *Per2*^−/−^ mice was not caused by impaired intestinal mucosal function. Even in *Per2*^−/−^ mice without prolapse, the expression of proinflammatory and adhesion factors is elevated compared to that in WT mice. This implies that inflammation in rectal tissue precedes and contributes to the development of rectal prolapse, and that prolapse in turn exacerbates inflammation. Proinflammatory cytokines, including TNF-α, IL-6, IL-1β, and IL-17, play an important role in drug-induced and spontaneous intestinal inflammation (38, 39, 40). The increase in these cytokines is accompanied by an increase in chemokines that recruit immune cells, including lymphocytes, granulocytes, and macrophages, which accumulate in the gut LP (41, 42). Mobilization of immune cells has been proposed as an early response of rectal tissue to defend against pathogen invasion (43). Despite slight differences in their composition and function, all types of macrophages share the common goal of eradicating pathogens (44). In *Per2*^−/−^ mice, populations of Th1, Th17, granulocytes, and macrophages at all three locations were significantly elevated, indicating the effort and inability of *Per2*^−/−^ mice to clear pathogens. This also suggests the possibility of immune dysfunction in *Per2*^−/−^ mice, which was previously unknown due to the lack of a well-defined immunophenotype.

Although some glandular/epithelial cells have potential regulatory roles in the rectal prolapse in *Per2*^*−/−*^ mice, macrophages should play a crucial role in the immune response to gut inflammation and in maintaining the stability of intestinal microbial structure by engulfing and digesting invading pathogens (45). *Per2*^−/−^ mice showed reduced phagocytosis, which may cause the inability of intestinal pathogens and harmful microorganisms to be removed, resulting in changes in gut microbiome. The capacity of macrophages to clear pathogens from the gut is essential for maintaining gut health and preventing chronic inflammation (46). Although gut macrophages and peripheral macrophages are not equivalent in function and properties (47), both types of cells belong to macrophages. Several studies have used the phagocytosis capacity of BMDM or macrophage cell lines to characterize the phagocytosis capacity of tissue macrophages. The effect of metabolites on BMDM phagocytosis has been used to assess phagocytosis and clearance of bacterial infections by resident intestinal macrophages (26). The phagocytic capacity of BMDM in young and old mice was compared to characterize the decreased clearance of *Streptococcus pneumoniae* in old mice (48). The phagocytic ability of mouse macrophage-like cells was used to evaluate the clearance ability of macrophages in heart and lung tissues (49, 50). In prolapsed *Per2*^−/−^ mice, impaired pathogen clearance of macrophages results in an increase in T cells, which in turn leads to the production of proinflammatory cytokines and the development of inflammation.

Macrophages require glycogen generation to store glycogen in their cells effectively and kill bacteria when stimulated (16, 51, 52). Glycogen in immune cells not only stores energy but also provides oxidative defense by producing NADPH and H_2_O_2_. Macrophages use G6P to synthesize glycogen and then degrade glycogen to produce G6P, which appears to be an overt futile glycogen synthesis/degradation cycle (16). PER2 binds to the transcription factor p65 to enhance GYS1 expression, promoting glycogen synthesis and pathogen clearance of macrophages after stimulation. The potential therapeutic implications of circadian regulation in immune function and inflammation warrant further investigation and may offer new avenues for the treatment of immune-related disorders.

In summary, our findings provide new evidence that the rhythmic gene *Per2* can determine the pathogen-clearing ability of macrophages by influencing glycogen synthesis. Our results could facilitate the identification of new treatments to control the increasingly crucial health problem of intestinal inflammation caused by aging and propose a mouse model that closely mimics human rectal prolapse.

## Experimental procedures

### Antibodies and reagents

The following antibodies were used in this study: anti-GYS1 (ab40810, abcam), anti-PER2 (NB100-125, Novus Bio), anti-PER2 (sc377290, Santa Cruz), anti-NFκB p65 (sc-8008, Santa Cruz), anti-NFκB p65 (ab16502, abcam), and anti-Flag (#14793, CST). ELISA kit for mouse was purchased from eBioscience. Actin-Tracker Red-Rhodamine (C2207S) was purchased from Beyotime. *Per2* shRNA lentiviral particles were purchased from Genepharma.

### Mice and animal studies

*Per2*^−/−^ mice used in this study were kindly provided by Dr C. Lee (53). *Per2*^−/−^ mice on the 129SvEv background were bred onto the C57BL/6J (Jackson Laboratory) background for 8 to 10 generations (N8-N10) according to standard genetic protocols. C57BL/6J mice were purchased from Model Animal Research Centre of Nanjing University. All mice were raised under standard humidity and controlled temperature conditions with a 12 h light to12 h dark cycle and were provided with free food and water. For consistent observations, all mice were male and all samples were collected at 7:00 PM unless specifically indicated. All animal care and use procedures were approved by the Institutional Animal Care and Use Committee of Nanjing University of Science and Technology (IACUC-NJUST-2019-0018). In the LPS-induced acute peritonitis mouse model, WT and nonprolapse *Per2*^−/−^ mice were intraperitoneally injected with 20 μg/kg body weight LPS. Spleen and rectum were separated 6 h later.

### Bacterial infection and quantification

LM was inoculated into brain–heart infusion broth (BD Biosciences) and cultured overnight at 37 °C (54). LM in midlogarithmic growth were collected, then resuspended in PBS and counted. WT and *Per2*^−/−^ mice were infected with 2 × 10^5^ LM intravenously (55). Spleen, liver, and kidney were taken on day 3 after infection and homogenized with 0.1% Trion X-100 PBS. Organ homogenates were subjected to serial dilutions and plated on brain–heart perfusion agar plates for measurement of colony-forming units as described previously.

### Phagocytosis and killing activity assay

The phagocytosis and killing activity assay *in vitro* was performed as described with minor modifications (56, 57). In brief, *E. coli* was taken and incubated at 60 °C for 1 h. The sterilized *E. coli* was coincubated with 1 μM 7-aminoactinomycin D at room temperature (RT) for 2 h. In order to enable *E. coli* to be phagocytosed by macrophages, the 7-aminoactinomycin D–stained *E. coli* was incubated with human serum at RT for 20 min. Opsonized *E. coli* was then added to 2.5 × 10^5^ macrophages and incubated in Roswell Park Memorial Institute-1640 culture medium containing 10% fetal bovine serum (FBS) at 37 °C for 1 h. Next, the samples were washed with cold PBS for three times and detected by flow cytometry.

*E. coli* were added to the BMDMs at multiplicity of infection of 1:10 and incubated for 2 h at 37 °C. The wells were washed with PBS to remove the unphagocytosed bacteria and fresh Dulbecco's modified Eagle's medium (DMEM) was added to the cells further for 2 h. After this 4-h phagocytosis period, infected BMDMs were washed with PBS and lysed using 0.05% Tween 20 to release internalized bacteria, and the cell lysates were diluted serially and plated on LB agar plates and then incubated overnight at 37 °C to count the number of colonies.

### Immunofluorescence stain

The entire rectum tissue was collected and immediately immunofluorescent stained as previously described (58). Briefly, the tissue was fixed with formalin, then dehydrated through ethanol, toluene, and finally embedded in paraffin. Slices of the rectums that were blocked in 2% bovine serum albumin (BSA) solution for 20 min, then incubated with anti-GYS1 (Abcam), anti-p65 (Abcam), and anti-PER2 (Santa Cruz) primary antibody at RT for 1 h. The sections were washed with PBS and incubated with secondary antibody CY3 Goat anti-rabbit and FITC Goat anti-rabbit (1:500) for 30 min under RT. The immunofluorescence staining of BMDMs was performed according to previous reports (16). Briefly, BMDMs were fixed with 4% paraformaldehyde for 20 min at RT after LPS stimulation and washed three times with PBS for 5 min per wash. After washing, the cells were blocked in 1% BSA at RT and then stained primary antibodies in 1% BSA at 4 °C overnight. The cells were then washed three times with 5% PBS-Tween (PBST) for 5 min per wash and stained with secondary antibody labeled with Alexa Fluor-488 and CY3 (1:1000, Abcam) for 1 h. After desiccation, the cells were counterstained with 4',6-diamidino-2-phenylindole (Sigma). Typical images of immunofluorescence sections were captured by fluorescence microscopy (Nikon).

### Histopathology analysis

Freshly isolated mouse tissues were quickly fixed with formalin, dehydrated, paraffin-embedded, and finally cut into 5-μm sections. The rectum and colon sections were stained with H&E and periodic acid–Schiff to analyze the severity of intestinal inflammation and observe the expression of MUC2. Histological analysis and percentage of positive staining area were performed using ImageJ 1.51j8 (https://imagej.nih.gov/ij/).

### Detection of glycogen

Tissue or cell glycogen content was determined using an anthrone reagent as previously described (59). Briefly, glycogen was extracted with a strong alkaline solution and determined with anthrone chromogenic reagent (Solarbio, BC0345) under strong acid conditions. The glycogen concentration was determined by measuring absorbance at 620 nm and standardized tissue weight or protein.

### Flow cytometry analysis

Single cells were isolated from spleens and MLNs according to previously reported methods (60). LP cells were isolated as described before (61). In brief, the rectal intestine was rinsed to remove fecal contents, then the rectum was cut into 0.5 cm fragments, transferred to a 50 ml centrifuge tube, and shaken at 37 °C for 30 min in Hank's balanced salt solution containing 10% FBS, 5 mM EDTA, and 10 mM Hepes. The cell suspension was passed through a cell filter. The remaining rectal tissue was chopped and transferred to DMEM containing 0.25 mg/ml VII collagenase, 0.125 U/ml Liberase TM, 10 mM Hepes (pH = 8), 0.1 M CaCl_2_, 0.05% DNase1, and 10% FBS. They were oscillated at 37 °C for 30 min. Cell suspensions were collected and passed through a cell strainer and then stained and analyzed. The cells were then stained with a combination of antibodies. The antibodies used were CD3, natural killer1.1, B220, IgD, CD3, CD4, CD8, IFN-γ, IL-17, CD11b, Ly6G, and F4/80. The analysis of cell subsets was performed by a NovoCyte flow cytometer (ACEA Bioscience Inc).

### RNA isolation and quantitative real-time PCR

Total RNA was extracted from freshly isolated colon, rectum, pancreas, and liver or cells using Karol reagent (Karoten). RNA was converted to complementary DNA using reverse transcript kits from Invitrogen. SYBR Green dye was used for Real-time PCR of complementary DNA, and the results were analyzed using an Applied Biosystems 7300 detection system. The relative gene expression in comparison with β-actin expression was calculated by the ΔΔCT method. The primers are shown in Table S1.

### Western blot analysis

Protein extraction from rectum tissues or cells was performed following the procedure described previously (62). The proteins were separated on 10% polyacrylamide gels by standard SDS/PAGE and then electrically transported to a polyvinylidene fluoride membrane. After blocking with 5% (w/v) skim milk powder dissolved in Tris-HCl buffered saline + Tween 20 at RT for 1 h, the membrane was incubated with primary antibody (anti-PER2, 1: 1000, Santa Cruz; anti-p65, 1: 1000, Abcam; anti-GYS1, 1: 10,000, Abcam; anti-β-actin, 1: 2000, Abcam) overnight at 4 °C, followed by incubation with an horseradish peroxidase-conjugated secondary antibody (1: 10,000). Signals were detected by a chemiluminescence detection system (Clinx Science Instrument).

### shRNAs preparation and lentiviruses infection

To generate *Per2* knockdown cell lines, Raw264.7 were infected with lentiviruses containing negative control shRNA or *Per2*-specific shRNA in the presence of 8 μg/ml polybrene and then selected with 2 μg/ml puromycin for 2 weeks as previously described. ShRNA directed against *Per2* mRNA were synthesized by Genepharma. Carrier type was LV3 (H1/GFP&Puro). The shRNA sequence is 5′-CACACTTGCCTCCGAAATA-3′.

### Gut microbiota analysis

The analysis of the gut microbiota by 16S rDNA amplicon sequencing was based on previously reported methods (63). Bacterial DNA was extracted from feces using a fecal Microbial Genomic DNA purification kit (Karoten), and 16S rDNA sequencing was performed by GENEWIZ. Degenerate PCR primers were used to amplify two highly variable regions of prokaryotic 16S rDNA, V3, and V4. The V3 and V4 regions were amplified using upstream primers containing the CCTACGGRRBGCASCAGKVRVGAAT sequence and downstream primers containing the GGACTACNVGGGTWTCTAATCC sequence. The original reads were screened and analyzed using UPARSE software (V7.0.1001, http://www.drive5.com/uparse/), and sequences with 97% homology were assigned to the same OTUs. Venn diagrams in OTUs and the relative abundance of species of different grades were drawn using the R package (http://cran.r-project.org/). QIIME software (v1.8.0, http://qiime.org/1.8.0/tutorials/index.html) 38 was used for sample clustering and diversity analysis. Linear discriminant analysis effect size analysis was used to determine the differentially abundant bacterial taxa between WT mice and nonprolapse *Per2*^−/−^ mice. In addition, to further verify the results of microbial diversity detection, partial representative mouse gut microbial genomic DNA were subjected to 16S rDNA amplification using SYBR Green PCR master mix (Applied Biosystems) to analyze the relative number of bacterial groups. The 16S rDNA primer sequences used for real-time PCR are shown in Table S2.

### Cell culture

The murine macrophage cell line Raw 264.7 cells were cultured in DMEM containing 25 mM glucose supplemented with 10% heat-inactivated FBS. All culture dishes were supplemented with 1% penicillin/streptomycin and maintained at 37 °C containing 5% CO_2_.

### Confocal microscopy

BMDMs were inoculated in confocal dishes and then treated desired. The cells were fixed in 4% paraformaldehyde for 10 min, and then incubated with 2% FBS for 1 h to block the nonspecific binding of the antibody. Incubate the appropriate primary antibody at 4 °C overnight. The cells were washed with PBST three times and incubated with the appropriate secondary antibodies (Invitrogen) for 1 h at RT without light exposure. Then, the cells were washed with PBST for three times, stained with 4',6-diamidino-2-phenylindole (Sigma), and washed with PBS. Nikon laser scanning confocal microscope (C2plus) was used for image acquisition.

### Preparation of mouse macrophages

Bone marrow cells isolated from WT and nonprolapse *Per2*^−/−^ mice were cultured in the complete Roswell Park Memorial Institute-1640 medium containing 10% L929 cell supernatant, 10% FBS, 10 mM glucose, 2 mM L-glutamine, and 100 U/ml penicillin-streptomycin for 5 days (64). On day 7, BMDMs were stimulated with 100 ng/ml LPS for 6 h or 24 h. Mouse peritoneal macrophages were collected by peritoneal lavage. Inject cold PBS into abdominal cavity, gently agitate, and remove. The peritoneal cell suspension was centrifuged at 1300 rpm, and the cells were mixed with 2 ml Red blood cell lysis buffer for 5 min at RT. The cells were washed and cultured on 6-well plates for 3 h, and the adherent cells were collected as peritoneal macrophages.

### Glucose consumption

Prior to LPS treatment, fresh complete medium was replaced, and a cell-free control group were set. After incubation for 6 or 24 h, the supernatant was collected and the glucose concentration was measured using a Glucose Assay kit according to the manufacturer's instructions. The glucose consumption rate was calculated as the glucose concentration in the supernatant minus that of the cell-free control group and normalized to the number of cells.

### Detection of NADPH, NADPH/NADP^+^, and H_2_O_2_

The NADPH content and NADPH/NADP^+^ ratio were determined by the NADP^+^/NADPH Quantification Colorimetric Kit (S0179, Beyotime). H_2_O_2_ levels were measured using the Hydrogen peroxide Content Detection Kit (BC3595, Solarbio). The tissues, cells, or culture medium supernatant were dissolved in 1 ml of ice-cold acetone, centrifuged at 8000*g* at 4 °C for 10 min, and the supernatant was collected and detected according to the manufacturer’s instructions.

### Plasmid constructs and transfection

The full-length complementary DNA encoding mouse p65 (Rela, NM_002247) was subcloned to p3XFLAG-CMV. All constructions were confirmed by DNA sequencing. NIH3T3 cells were transfected instantaneously using Lipofectamine 2000 (Invitrogen). The experiment was conducted 24 h after transfection.

### PER2/P65 interaction analysis

Analysis of PER2/P65 complex regulation of Gys1 transcription by co-IP and ChIP is based on previously reported methods with slight modification (65). For co-IP, BMDMs were homogenized and lysed with a Non-denaturing lysis buffer containing 20 mM Tris–HCl pH 8.0, 137 mM NaCl, 2 mM EDTA, and 1% NP-40 with protease inhibitor cocktail. We then incubated lysates with antibody (anti-PER2, Santa Cruz, sc377290; anti-NFκB p65, Santa Cruz, sc-8008) overnight at 4 °C, incubating with Protein G-sepharose 4B (Invitrogen). Immunoprecipitates were washed three times with wash buffer containing 10 mM Tris–HCl pH 7.4, 150 mM NaCl, 1 mM EDTA, 1% Triton X-100, 0.2 mM sodium orthovanadate with protease inhibitor cocktail and boiled in SDS-PAGE loading buffer. Proteins were analyzed by Western blotting.

The ChIP assay was performed as previously described with some modifications (30). Briefly, the cells were collected and crosslinked in 1% formaldehyde for 15 min. Crosslinking was stopped with 0.125 M glycine, and samples were rinsed with 1× PBS containing 1 mM PMSF. The nuclei were pelleted and resuspended in lysis buffer (1% SDS, 10 mM EDTA, 50 mM Tris–HCl, pH 8.1, 0.8 μg/ml pepstatin A, 0.6 μg/ml leupeptin, 1 mM PMSF). The suspension was sonicated using a sonicator (KS-130, Ningbo Kaisheng) to generate DNA fragments averaging 400 to 500 bp in length and clarified by centrifugation. For immunoprecipitation, the supernatant was diluted 10-fold with dilution buffer (1% Triton X-100, 2 mM EDTA, 150 mM NaCl, 20 mM Tris–HCl, pH 8.1) and divided into fractions for control immunoglobulin G and anti-p65 or anti-PER2. Protein A-sepharose beads preblocked with 300 μg/ml sheared salmon sperm DNA, 0.1% BSA was used to precipitate antibody–chromatin complexes. Beads were washed sequentially for 5 min each with Tris-SDS-EDTA buffer containing 150 and 500 mM NaCl, buffer III, and Tris-EDTA buffer (pH 8). Immunocomplexes were eluted, and the elution was heated to reverse the formaldehyde crosslinks and phenol: chloroform extracted. For PCR analysis of the ChIP samples, purified immunoprecipitates were dissolved in 20 μl water. The primers are shown in Table S3.

### Enzyme-linked immunosorbent assay

ELISA was performed to detect the secreted cytokines in culture supernatants of rectum explant cultures. The differentiated cells were restimulated with 50 ng/ml phorbol 12-myristate 13-acetate and 1 μg/ml ionomycin for 4 h, and ELISA was performed from culture supernatants. Cytokines production in the supernatants were quantified by ELISA kits according to the manufacturer’s protocol.

### Statistical analysis

Data were presented as means ± SD. Statistical analysis was performed by Student’s *t* test or multifactorial ANOVA, followed by Tukey’s post hoc test with the GraphPad Prism 8 (GraphPad Software, https://www.graphpad.com/). *p* < 0.05 was considered statistically significant; *p* value results were denoted by asterisks in the figures (∗*p* < 0.05, ∗∗*p* < 0.01).

## Ethics approval

All animal experiments complied with the ARRIVE guidelines were approved and conducted in accordance with the Animal Care and Use Committee of Nanjing University of Science and Technology.

## Data availability

All data relevant to the study are included in the article or uploaded as supporting information. Datasets generated and analyzed during this study are available from the corresponding author on reasonable request.

## Supporting information

This article contains supporting information (66).

## Conflict of interest

J. Z. is the guarantor of this work and, as such, had full access to all the data in the study and take responsibility for the integrity of the data and the accuracy of the data analysis. The other authors declare that they have no conflicts of interest with the contents of this article.
