# Supplementary material for: PER2/P65-driven glycogen synthase 1 transcription in macrophages modulates gut inflammation and pathogenesis of rectal prolapse
Source: J Biol Chem. 2023 Sep 1;299(10):105219. doi: 10.1016/j.jbc.2023.105219 (PMC10534228; doi:10.1016/j.jbc.2023.105219)
Supplement: Supporting Information [file mmc1.docx]

**PER2/p65-driven glycogen synthase 1 transcription in macrophages modulates gut inflammation and pathogenesis of rectal prolapse**

Zhao Ding^1#^, Wenhao Ge^1#^, Xiaodong Xu^1^, Xi Xu^1^, Shiming Wang^1^ and Jianfa Zhang^1*^

**List:**

Supplementary Experimental procedures;

Supplementary Figure 1;

Supplementary Figure 2;

Supplementary Figure 3;

Supplementary Figure 4;

Supplementary Figure 5;

Supplementary Figure 6;

Supplementary Figure 7;

Supplementary Figure 8;

Supplementary Figure 9;

Supplementary Table S1;

Supplementary Table S2;

Supplementary Table S3.

**Supplementary Experimental procedures**

**Immunohistochemistry staining**

Rectal tissue was fixed with paraformaldehyde, embedded in paraffin, sliced (5 μm), and transferred to slides. The sections were washed 3 times in PBS, treated with 3% H_2_O_2_ in PBS for 10 minutes, blocked with 10% BSA in PBS for 1 hour, and incubated with a primary antibody against CD11b, F4/80, and Ly6G overnight. The secondary antibody was then placed at RT for 30 min. Then streptavidin was labeled with diluted HRP and incubated at 37°C for 30 min. Finally, color was developed with DAB solution. Typical images of immunohistochemistry sections were captured by microscopy (Nikon, Tokyo, Japan).

**Cell viability assay**

Raw264.7 cells in the negative control (NC) group and Sh*per2* group were plated into 96-well culture plates. The cells were then cultured in the absence or presence of 100 ng/mL LPS for 24 h. After 50 μL MTT (1 mg/mL) solutions added to each well for 4 h, the formazan crystals were obtained by centrifugation at 300 g for 10 min. The optical density was measured at 570 nm by Microplate reader (Bio-Tek, USA) following the removal of supernatant and dissolution in the DMSO.

**Mitochondrial reactive oxygen species (Mit-ROS) analysis**

Cellular mitochondrial ROS generation was detected using a mitochondrial ROS-specific staining kit (Bestbio, China). Cells were treated with 1 mL fluorescent probe and incubated at 37 °C for 30 min. Flow cytometry analysis was performed immediately after washing in PBS and scraping the cells.

**Pyruvate and nitric oxide (NO) assay**

Pyruvate Assay Kit (BC2205, Solarbio, China) and NO Assay Kit (BC1470, Solarbio, China) were used to measure the concentration of pyruvate and NO levels. After treating macrophages with LPS for 24 hours, the cells were collected and followed the product instructions. The relative absorbance was measured by spectrophotometer.

**Chromatin Immunoprecipitation**

Chromatin immunoprecipitation (ChIP) was used to analyze the binding of PER2 and p65 at the promoter region of pro-inflammatory cytokines (*tnf-α*, *il-1β*, and *il-6*), as previously described (1). The primers are shown in Supplementary Table S3.

**
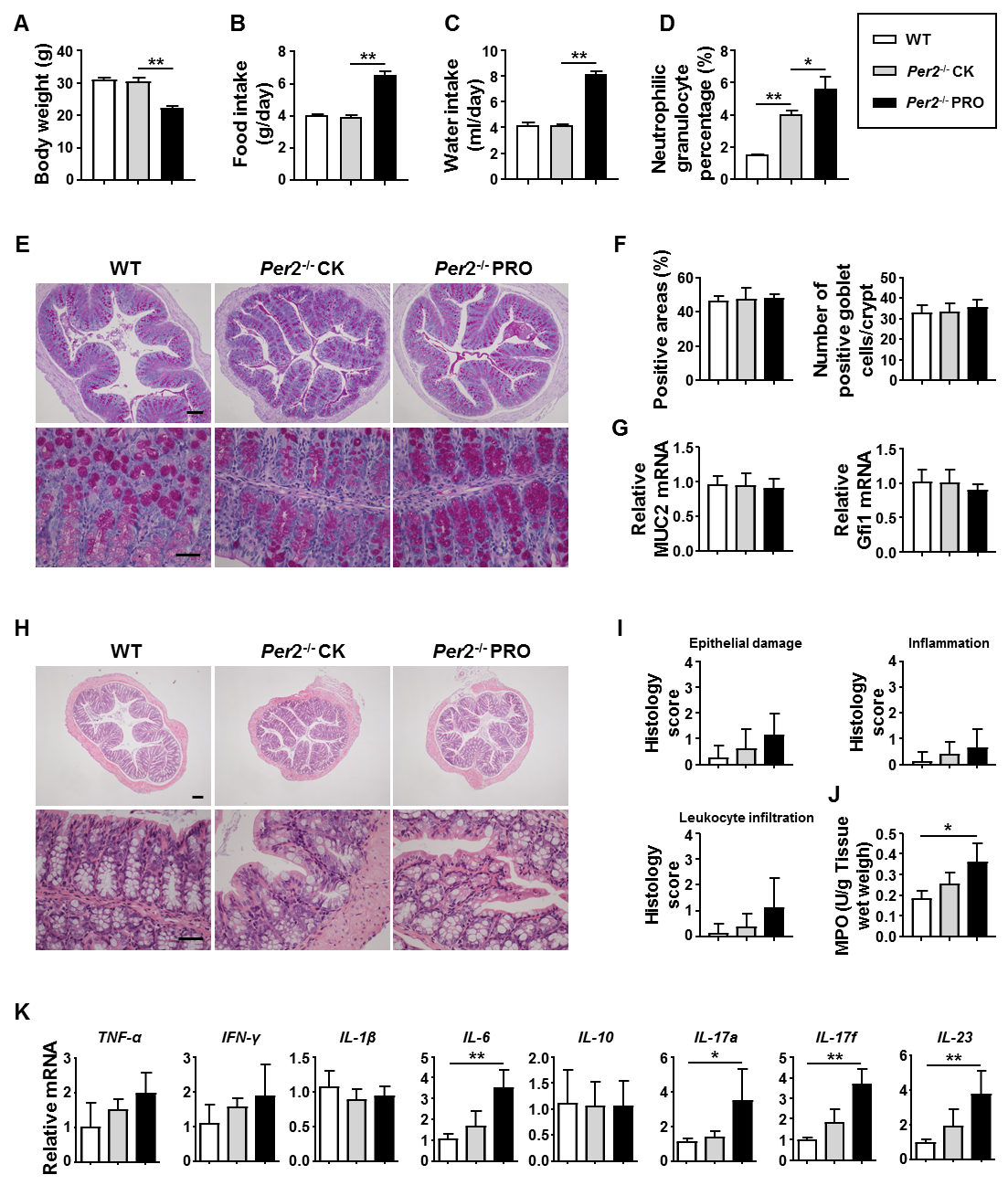
**

**Supplementary Figure 1. *Per2*^-/-^ mice developed milder inflammation in the colon, related to Fig. 1.**

(A) Body weight of 16-week-old male WT mice, non-prolapsed and prolapsed *Per2*^-/-^ mice. (B) Food intake. (C) Water intake. (D) The proportion of neutrophilic granulocyte in the blood. (E) Periodic acid Schiff (PAS)-stained sections of colons from WT mice, non-prolapsed and prolapsed *Per2*^-/-^ mice. Scale bars, 100 μm (top row) or 10 μm (bottom row). (F) Assessment of positive areas and number of positive goblet cells/crypt of PAS-stained sections. (G) The mRNA expression levels of *Muc2* and *Gfi1* in the colons were measured by real-time PCR. (H) H&E-stained sections of colons from WT mice, non-prolapsed and prolapsed *Per2*^-/-^ mice. Scale bars, 100 μm (top row) or 10 μm (bottom row). (I) H&E-stained sections were scored for epithelial damage, inflammation and leukocyte infiltration. Data are from one experiment representative of three independent experiments with similar results. (J) Colons myeloperoxidase (MPO) activity of WT mice, non-prolapsed and prolapsed *Per2*^-/-^ mice. (K) The mRNA expression levels of cytokines (*Tnf-α*, *Ifn-γ*, *il-1β*, *il-6*, *il-10*, *il-17a*, *il-17f* and *il-23*) in the colons were measured by real-time PCR. WT, WT mice; *Per2*^-/-^ CK, non-prolapsed *Per2*^-/-^ mice; *Per2*^-/-^ PRO, prolapsed *Per2*^-/-^ mice. Data were shown as mean ± SD. Statistical analysis was performed by one-way ANOVA. Data are pooled from four independent experiments (A-D; of n = 6-8 mice) or are from one experiment representative of three independent experiments with similar results (E-J). *p<0.05, **p<0.01.


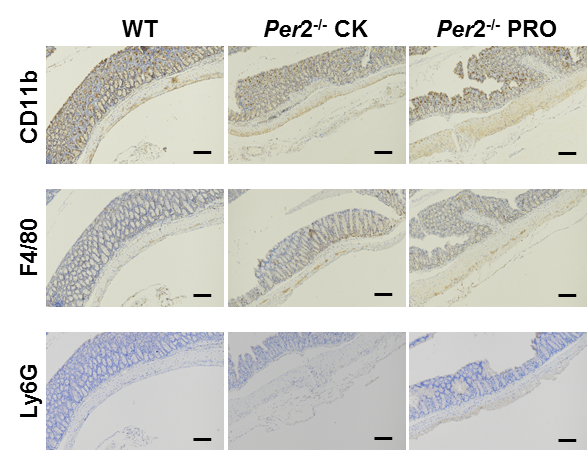


**Supplementary Figure 2. *Per2*^-/-^ mice had increased infiltration of neutrophils and macrophages in rectal tissue, related to Fig. 2.**

Immunohistochemistry of CD11b, F4/80, and Ly6G in rectums from WT mice, non-prolapsed and prolapsed *Per2*^-/-^ mice. Scale bars, 100 μm.


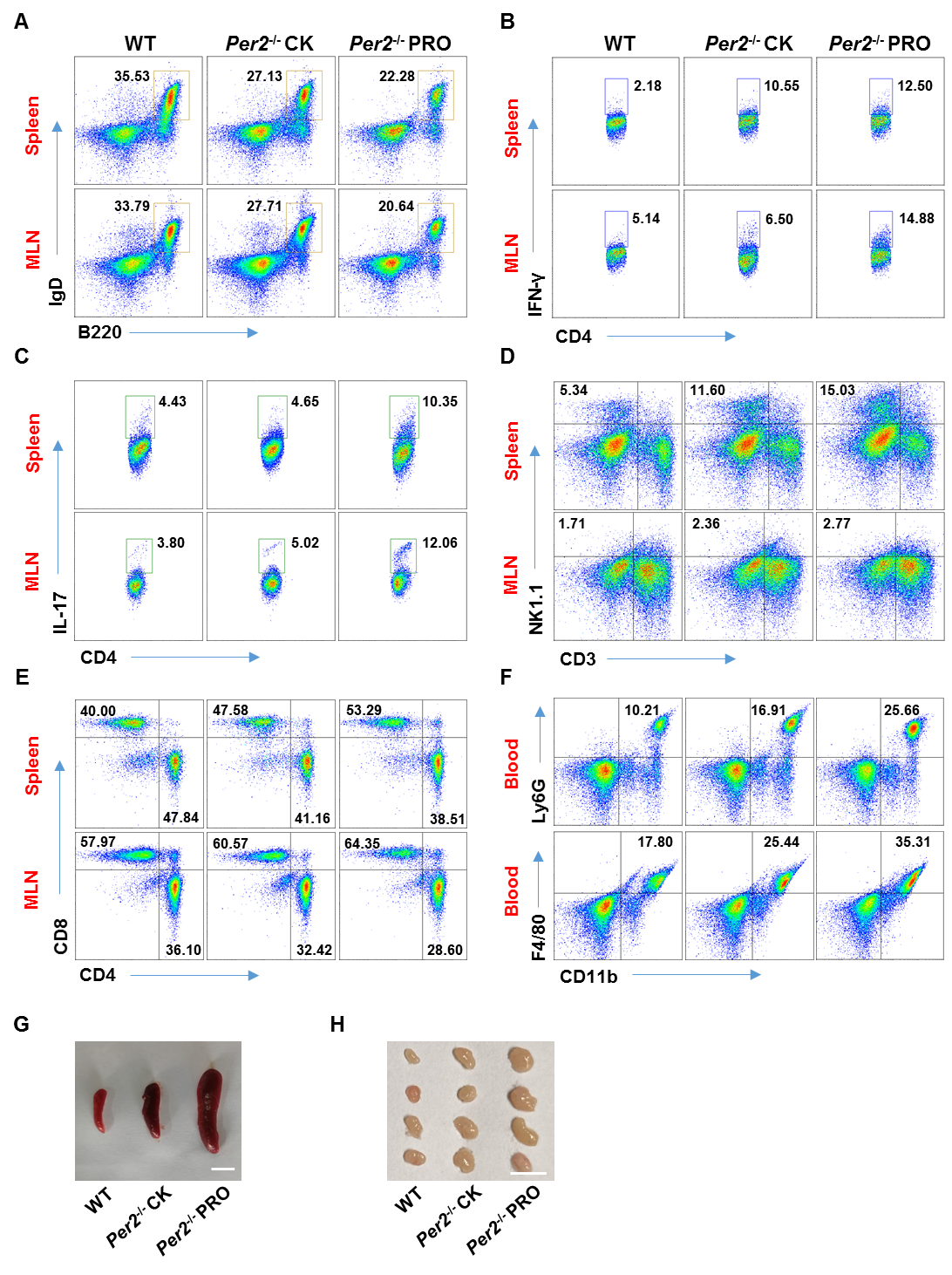


**Supplementary Figure 3. *Per2*^-/-^ mice had altered immune configuration in spleen and MLN, related to Fig. 2.**

(A-E) Flow cytometry of spleen and mesenteric lymph nodes (MLN) isolated from WT mice, non-prolapsed and prolapsed *Per2*^-/-^ mice (n = 5-6). (A) Single cells stained with antibodies against CD3, B220 and IgD. Numbers adjacent to outlined areas indicate percent CD3^-^ B220^+^ IgD^+^ cells (mature IgD^+^ B cells). (B) Single cells stained with antibodies against CD3, CD4 and IFN-γ. Numbers adjacent to outlined areas indicate percent CD4^+^ IFN-γ^+^ cells (Th1 cells). (C) Single cells stained with antibodies against CD3, CD4 and IL-17. Numbers adjacent to outlined areas indicate percent CD4^+^ IL-17^+^ cells (Th17 cells). (D) Single cells stained with antibodies against CD3 and NK1.1. Numbers indicate percent CD3^-^ NK1.1^+^ cells (NK cells). (E) Single cells stained with antibodies against CD3, CD4 and CD8. Numbers indicate percent CD4^+^ T cells and CD8^+^ T cells. (F) Immune cells from the blood stained with antibodies against CD11b, Ly6G and F4/80. Numbers indicate percent CD11b^+^ Ly6G^+^ cells (neutrophils) (top row) or CD11b^+^ F4/80^+^ cells (macrophages) (bottom row). (G-H) Size of the spleen and the MLNs of the WT mice, non-prolapsed and prolapsed *Per2*^-/-^ mice. Scale bars, 0.5 cm.


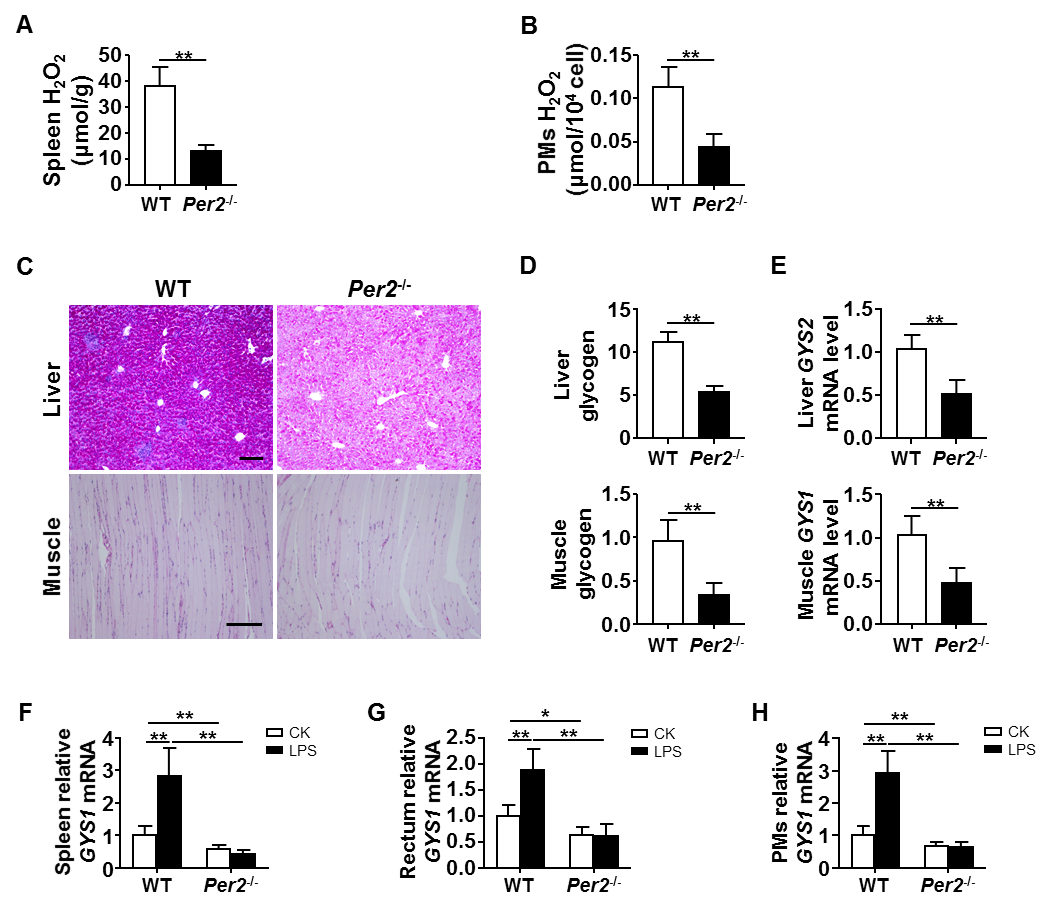


**Supplementary Figure 4. H_2_O_2_ production in *Per2*^-/-^ mice was reduced after LPS stimulation, related to Fig. 4.**

(A-B) 6-week-old WT and *Per2*^-/-^ mice were infected with *Listeria monocytogenes* (LM), and the spleen and peritoneal macrophages (PMs) were separated for hydrogen peroxide (H_2_O_2_) determination 3 days later (n = 6-8). (A-B) H_2_O_2_ levels in the spleen (A) and PMs (B) of LM-challenged WT and *Per2*^-/-^ mice. (C-E) Liver and muscle were separated from 16-week-old WT and *Per2*^-/-^ mice (n = 5-6). (C) PAS-stained sections of liver and muscle. Scale bars, 100 μm. (D) Glycogen levels in liver and muscle. (E) Relative mRNA expression of *Gys2* or *Gys1* of liver and muscle. (F-H) 6-week-old WT and *Per2*^-/-^ mice were challenged intraperitoneally with 5 mg LPS/kg in ZT12 (n = 6-8). Spleen, rectum and PMs were separated for real-time PCR 6 hours later. Relative mRNA expression of *Gys1* of spleen (F), rectum (G) and PMs (H). Data are pooled from three independent experiments. Data were shown as mean ± SD. Statistical analysis was performed by Student’s *t*-test and two-way ANOVA. *p<0.05, **p<0.01.


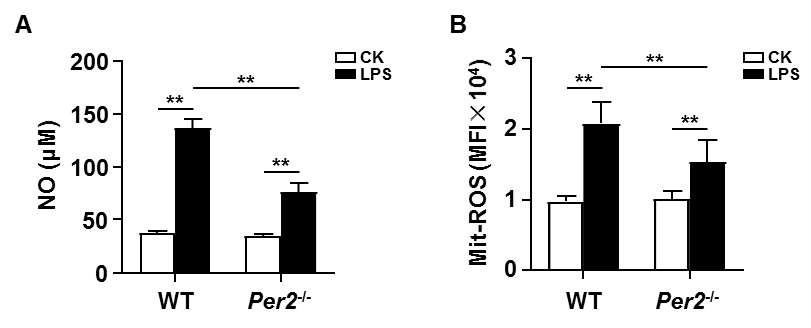


**Supplementary Figure 5. The deficient of *Per2* reduces nitric oxide and mitochondrial reactive oxygen species, related to Fig. 4.**

(A-B) BMDMs from WT and *Per2*^-/-^ mice were stimulated with LPS for 24 h. The intracellular nitric oxide (A) and mitochondrial reactive oxygen species (Mit-ROS) (B) were analyzed. MFI, mean fluorescence intensity. Data are pooled from three independent experiments. Data were shown as mean ± SD. Statistical analysis was performed by two-way ANOVA. **p<0.01.


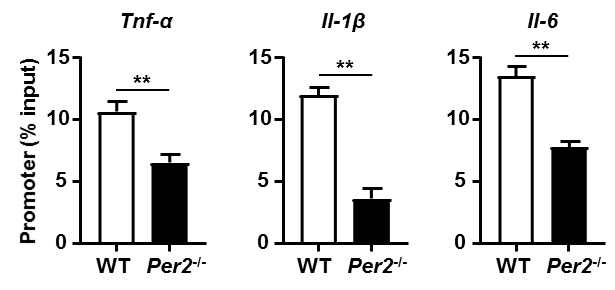


**Supplementary Figure 6. *Per2* deficiency resulted in diminished binding of p65 to *tnf-α*, *il-1β*, and *il-6* promoters, related to Fig. 6.**

The promoters of *tnf-α*, *il-1β*, and *il-6* bound to p65 were quantitatively detected by ChIP-qPCR after LPS stimulation of BMDMs from WT and *Per2*^-/-^ mice. Data were shown as mean ± SD. Statistical analysis was performed by Student’s *t*-test. **p<0.01.


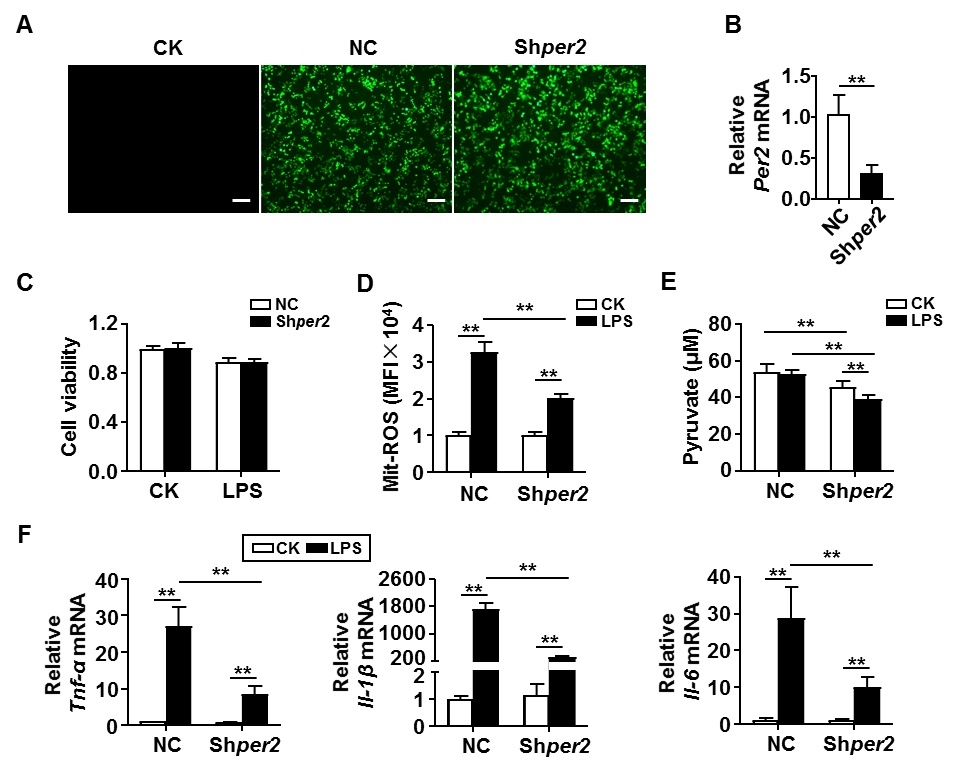


**Supplementary Figure 7. Inhibition of *Per2* reduced the inflammatory response in Raw264.7 cells, related to Fig. 7.**

(A) Representative ﬂuorescent images of the intracellular distribution of GFP shRNA. Pictures taken at original magniﬁcation (× 40). Scale bars, 100 μm. (B) Quantitative RT-PCR analysis of *Per2* mRNA in the Sh*Per2*-treated Raw264.7 cells. (C-F) Raw264.7 cells in the negative control (NC) group and Shper2 group were stimulated with or without 100 ng/mL LPS for 24 h. Cell viability (C), mitochondrial reactive oxygen species (Mit-ROS) (D) and pyruvate (E) levels of Raw264.7 cells were analyzed. (F) Relative mRNA expression of *Tnf-α, il-1β*, and *il-6* were determined by real-time PCR. MFI, mean fluorescence intensity. Data are pooled from three independent experiments. Data were shown as mean ± SD. Statistical analysis was performed by Student’s *t*-test and two-way ANOVA. *p<0.05, **p<0.01.


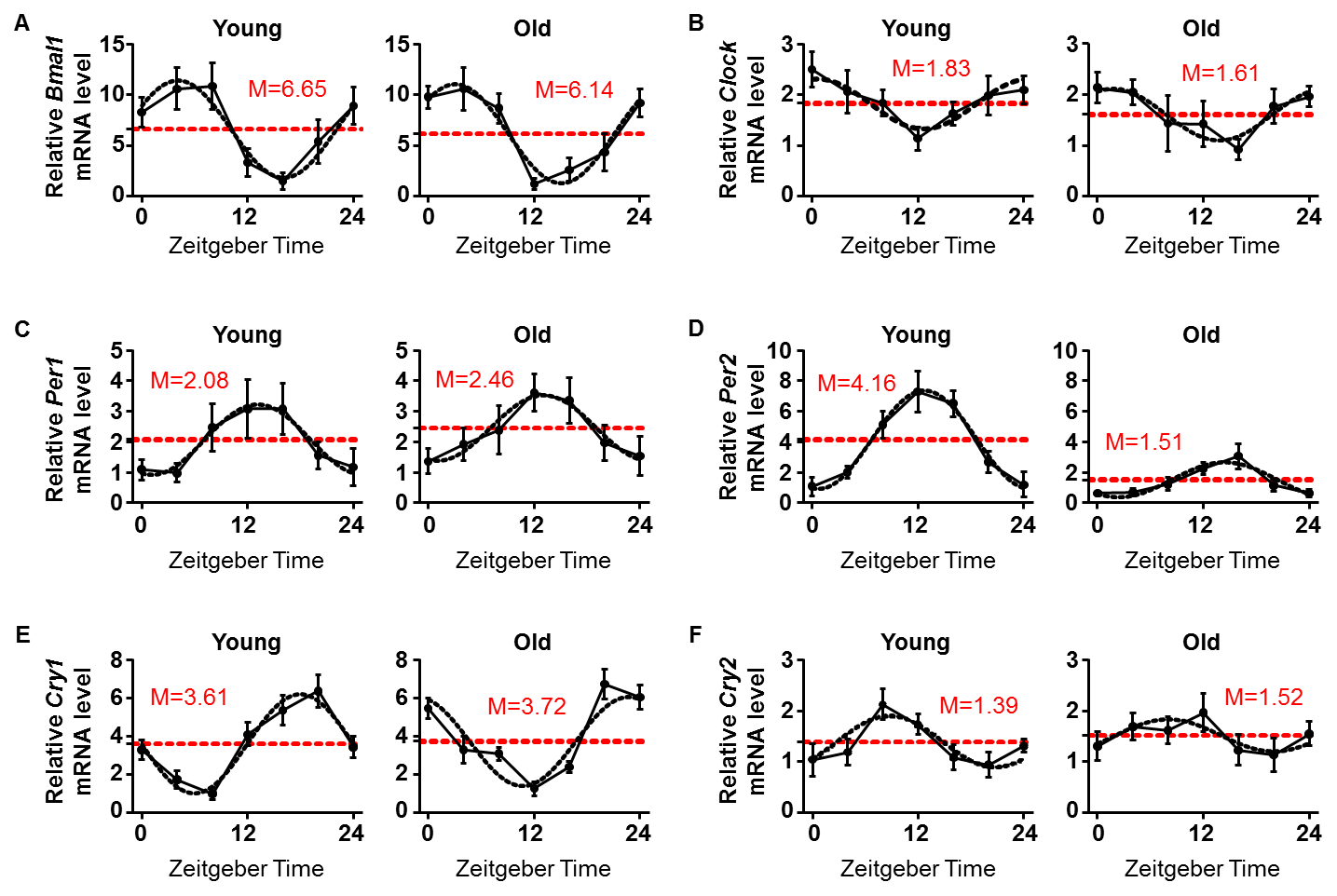


**Supplementary Figure 8. *Per2* circadian rhythm expression was impaired in the rectum of old mice.**

(A-F) The 8-week-old male mice were taken as the young group and the 64-week-old male mice as the old group. The circadian expression of six core clock genes in rectum was detected by real-time PCR. Diurnal mRNA levels of *Bmal1* (A), *Clock* (B), *Per1* (C), *Per2* (D), *Cry1* (E) and *Cry2* (F) in the rectum of young (8 weeks of age) and old (64 weeks of age) mice. Solid lines represent experimental response curves. Dashed lines represent fit cosine curves. Solid horizontal lines represent the circadian Mesors (24-h averages). All data were expressed as mean ± SD (n = 4 at each time point).


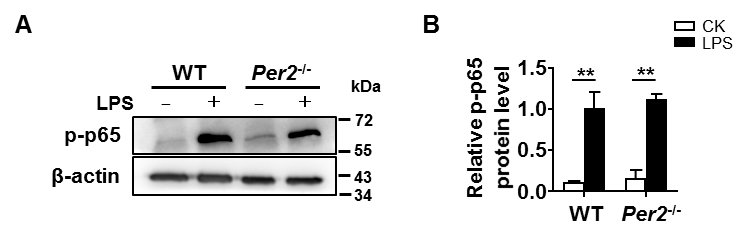


**Supplementary Figure 9. The deficient of *Per2* does not affect phosphorylation of p65.**

(A-B) BMDMs from WT and *Per2*^-/-^ mice were stimulated with LPS for 24 h. (A) p-p65 expression were determined by western blot. (B) Fluorescence intensity of p-p65 was analyzed. Data were shown as mean ± SD. Statistical analysis was performed by two-way ANOVA. *p<0.05, **p<0.01.

Supplementary Table S1 Primer sequences used in quantitative RT-PCR analysis

| Gene | Primer Sequences |
| --- | --- |
| *Muc2* | Forward: 5'- GCTGACGAGTGGTTGGTGAATG-3'  Reverse: 5'- GATGAGGTGGCAGACAGGAGAC-3' |
| *Gfi1* | Forward: 5'- TGGGCGGCGGCTCCTACAAAT-3'  Reverse: 5'- ACGCCACACCTCTTGTGGACT-3' |
| *Ccl2* | Forward: 5'- TTAAAAACCTGGATCGGAACCAA-3'  Reverse: 5'- GCATTAGCTTCAGATTTACGGGT-3' |
| *Ccl5* | Forward: 5'- TCGAGTGACAAACACGACTGC-3'  Reverse: 5'- GCTGCTTTGCCTACCTCTCC-3' |
| *Ccl7* | Forward: 5'- GCTGCTTTCAGCATCCAAGTG-3'  Reverse: 5'- CCAGGGACACCGACTACTG-3' |
| *Ccl20* | Forward: 5'- GTGGGTTTCACAAGACAGATG-3'  Reverse: 5'- TTTTCACCCAGTTCTGCTTTG-3' |
| *Cxcl1* | Forward: 5'- CTGGGATTCACCTCAAGAACATC-3'  Reverse: 5'- CAGGGTCAAGGCAAGCCTC-3' |
| *Cxcl2* | Forward: 5'- TCAAGAACATCCAGAGCTTGAG-3'  Reverse: 5'- TTCAGGGTCAAGGCAAACTT-3' |
| *Cxcl5* | Forward: 5'- GGTCCACAGTGCCCTACG-3'  Reverse: 5'- GCGAGTGCATTCCGCTTA-3' |
| *Cxcl8* | Forward: 5'- GCTGGGATTCACCTCAAGAA-3'  Reverse: 5'- CTTTTGGACAATTTTCTGAACCA-3' |
| *TNF-α* | Forward: 5'- GCCTCTTCTCATTCCTGCTT-3'  Reverse: 5'- CACTTGGTGGTTTGCTAGGA-3' |
| *IFN-γ* | Forward: 5'- AGGAACTGGCAAAAGGATGGTG-3'  Reverse: 5'- GTGCTGGCAGAATTATTCTTATTG-3' |
| *IL-1β* | Forward: 5'- GAGCTTCAGGCAGGCAGTATC-3'  Reverse: 5'- GTATAGATTCTTTCCTTTGAGGC-3' |
| *IL-6* | Forward: 5'- TTCCATCCAGTTGCCTTCTT-3'  Reverse: 5'- ATTTCCACGATTTCCCAGAG-3' |
| *IL-10* | Forward: 5'- GAGGCTACGGCGCTGTCAT-3'  Reverse: 5'- CCACGGCCTTGCTCTTGTT-3' |
| *IL-17a* | Forward: 5'- CTCAGACTACCTCAACCGTTCC-3'  Reverse: 5'- ATGTGGTGGTCCAGCTTTCC-3' |
| *IL-17f* | Forward: 5'- CATACCCAGGAAGACATACTTAGAAG-3'  Reverse: 5'- AGTCCCAACATCAACAGTAGC-3' |
| *IL-23* | Forward: 5'- TCCGTTCCAAGATCCTTCGA-3'  Reverse: 5'- TGTTGGCACTAAGGGCTCAG-3' |
| *Pygl* | Forward: 5'- GGCAGAAGTGGTGAACAATGACC-3'  Reverse: 5'- TCCGATAGGTCTGTGGCTGGAA-3' |
| *Gys1* | Forward: 5'- CACAGAACGGTTGTCGGACTTG-3'  Reverse: 5'- AGGTGAAGTGGTCTGGAAAGGC-3' |
| *Gys2* | Forward: 5'- GCCAGACACCTGACACTGA-3'  Reverse: 5'- TCCGTCGTTGGTGGTGATG-3' |
| *p65* | Forward: 5'- CGCGGATCCGCCACCATGGACGAACTG-3'  Reverse: 5'- CCGCTCGAGTTAGGAGCTGATCTG-3' |
| *Bmal1* | Forward: 5'- AGGTCGAATGATTGCTGAGG-3'  Reverse: 5'- TCTTCTTGCCTCCTGGAGAA-3' |
| *Clock* | Forward: 5'- ACAGCAGTGGATTTGGCTTCA-3'  Reverse: 5'- TTCTCGTCGTCTTTCAGCCCT-3' |
| *Per1* | Forward: 5'- GCCAACCAGGAGTTCTACCA-3'  Reverse: 5'- CCTGTCAGGAAGGAGACAGC-3' |
| *Per2* | Forward: 5'- CTCAAGTACGCCCTGAGGAG-3'  Reverse: 5'- GGCCACTTGGTCAGAGATGT-3' |
| *Cry1* | Forward: 5'- AGATGGCTTACCCTCTGCAGTG-3'  Reverse: 5'- CTTTCAAAGTTTGCCACCCAAG-3' |
| *Cry2* | Forward: 5'- ATGCCCCAGAGTCCATTGAGAA-3'  Reverse: 5'- TGAGGTCTTCCACACAGGAAGG-3' |
| *β-actin* | Forward: 5'- TGCGTGACATCAAAGAGAAG-3'  Reverse: 5'- CGGATGTCAACGTCACACTT-3' |

Supplementary Table S2 Primer sequences of bacteria

| Bacteria | Sequences |
| --- | --- |
| *Helicobacter* | Forward: 5'-ATGGGTAAGAAAATAGCAAA-3'  Reverse: 5'-CTATTTCATATCCATAAGCT-3' |
| *Lactococcus* | Forward: 5'- GCCACATTGGGACTGAGACA-3'  Reverse: 5'- GTTTACGGCGTGGACTACCA-3' |
| *Bacteroides* | Forward: 5'- GGTTCTGAGAGGAGGTCCC-3'  Reverse: 5'- CTGCCTCCCGTAGGAGT-3' |
| *Ruminococcus_1* | Forward: 5'- GAAAGCCTGATGCAGCGATG-3'  Reverse: 5'- CCTGGTAAGGTTCTTCGCGT-3' |
| *Candidatus_*  *Saccharimonas* | Forward: 5'- GCAAGCGAGTCTGCAACAAA-3'  Reverse: 5'- TTTTCTGCCAACCGCCTTTG-3' |
| *Rikenella* | Forward: 5'- CGGAAGTCTGAACCAGCCAT-3'  Reverse: 5'- TAAACCACATGTCCCTCCGC-3' |
| *Ruminococcaceae* | Forward: 5'- GGGGAATATTGCACAATGGAGG-3'  Reverse: 5'- GTTTGCTCCCCACGCTTTC-3' |
| *Prevotellaceae_*  *UCG-001* | Forward: 5'- CCAGCCAAGTAGCGTGCA-3'  Reverse: 5'- TGGACCTTCCGTATTACC-3' |
| *Lactobacillus* | Forward: 5'- AGCAGTAGGGAATCTTCCA-3'  Reverse: 5'- CACCGCTACACATGGAG-3' |
| *Blautia* | Forward: 5'- GTGAAGGAAGAAGTATCTCGG-3'  Reverse: 5'- TTGGTAAGGTTCTTCGCGTT-3' |
| *Parasutterella* | Forward: 5'- AACGTRTCCGCTCGTGGGGGAC-3'  Reverse: 5'- CGGAATAGCTGGATCAGGCTTG-3' |
| *Adlercreutzia* | Forward: 5'- ACGGTACCTGCAGAAGAAGC-3'  Reverse: 5'- CCTGGTAAGGTTCTTCGCGTT-3' |
| *Eubacteria* | Forward: 5'- CCTACGGGAGGCAGCAG-3'  Reverse: 5'- ATTACCGCGGCTGCTGG-3' |

Supplementary Table S3 Primer sequences used in ChIP analysis

| Site | Primer Sequences |
| --- | --- |
| *Gys1-1* | Forward: 5'-GGGAGCCTTCACCTTCCTCT-3'  Reverse: 5'-AGAGCATGGGGTCTTGTCCA-3' |
| *Gys1-2* | Forward: 5'-AGCTAGTGCCAGGCCCAATA-3'  Reverse: 5'-CCAGAGCCTGGAGTGACAGA-3' |
| *Gys1-3* | Forward: 5'-TCCTCCCAACACTTCCCACA-3'  Reverse: 5'-GCATCATGGCAACCGTGGTA-3' |
| *Gys1-4* | Forward: 5'-AGGAGCGCGGTCTTTATCCT-3'  Reverse: 5'-ATCGTGGAGGACGTGTGGTT-3' |
| *Gys1-5* | Forward: 5'-GGTTCCTGACGTCTCTTGCTCC-3'  Reverse: 5'-ACTGCCCACCCTGCTGGTCTA-3' |
| *tnf-α* | Forward: 5'-GAGGCAATAGGTTTTGAGG-3'  Reverse: 5'-AAGCATCAAGGATACCCTC-3' |
| *il-1β* | Forward: 5'-ACCCTCACCCTCCAACAAAG-3'  Reverse: 5'-TGGAAGGGCAAGGAGTAGCA-3' |
| *il-6* | Forward: 5'-GTGTCTTCCACTTTGTCCCACA-3'  Reverse: 5'-TGGAAGGGCAAGGAGTAGCA-3' |
| *Gapdh* | Forward: 5'-GTGGGTGCGTGTAAGCCTCTA-3'  Reverse: 5'-TGTCGCTGCCTGCATCAG-3' |

**References**

1. Bao H, Qu Q, Zhang W, Wang X, Fang J, Xue J, et al. NRF2 Exerts Anti-Inflammatory Effects in LPS-Induced gEECs by Inhibiting the Activation of the NF-κB. Mediators Inflamm. 2021;2021:9960721.
